# Age dependent changes in the LPS induced transcriptome of bovine dermal fibroblasts occurs without major changes in the methylome

**DOI:** 10.1186/s12864-015-1223-z

**Published:** 2015-01-27

**Authors:** Benjamin B Green, Stephanie D McKay, David E Kerr

**Affiliations:** Department of Animal Science, University of Vermont, Terrill Hall, 570 Main Street, Burlington, VT 05405 USA

**Keywords:** Epigenetics, MIRA-Seq, RNA-Seq, LPS, TLR4

## Abstract

**Background:**

By comparing fibroblasts collected from animals at 5-months or 16-months of age we have previously found that the cultures from older animals produce much more IL-8 in response to lipopolysaccharide (LPS) stimulation. We now expand this finding by examining whole transcriptome differences in the LPS response between cultures from the same animals at different ages, and also investigate the contribution of DNA methylation to the epigenetic basis for the age-dependent increases in responsiveness.

**Results:**

Age-dependent differences in IL-8 production by fibroblasts in response to LPS exposure for 24 h were abolished by pretreatment of cultures with a DNA demethylation agent, 5-aza-2′deoxycytidine (AZA). RNA-Seq analysis of fibroblasts collected from the same individuals at either 5 or 16 months of age and exposed in parallel to LPS for 0, 2, and 8 h revealed a robust response to LPS that was much greater in the cultures from older animals. Pro-inflammatory genes including IL-8, IL-6, TNF-α, and CCL20 (among many other immune associated genes), were more highly expressed (FDR < 0.05) in the 16-month old cultures following LPS exposure. Methylated CpG island recovery assay sequencing (MIRA-Seq) revealed numerous methylation peaks spread across the genome, combined with an overall hypomethylation of gene promoter regions, and a remarkable similarity, except for 20 regions along the genome, between the fibroblasts collected at the two ages from the same animals.

**Conclusions:**

The fibroblast pro-inflammatory response to LPS increases dramatically from 5 to 16 months of age within individual animals. A better understanding of the mechanisms underlying this process could illuminate the physiological processes by which the innate immune response develops and possibly individual variation in innate immune response arises. In addition, although relatively unchanged by age, our data presents a general overview of the bovine fibroblast methylome as a guide for future studies in cattle epigenetics utilizing this cell type.

**Electronic supplementary material:**

The online version of this article (doi:10.1186/s12864-015-1223-z) contains supplementary material, which is available to authorized users.

## Background

The innate immune response plays an important role in the clearance of pathogens following an infection, including during bovine mastitis. Components of the recognition and response pathway, mediated by toll-like receptor (TLR)4, are susceptible to regulation of gene expression by epigenetic mechanisms. For example, DNA-methylation of the *TLR4* gene promoter has been linked to lower expression and diminished response to LPS in intestinal epithelial cells [1]. Conversely, DNA hypomethylation has been implicated in over expression of *myeloid differentiation factor (MD)-2* in human IECs leading to higher responsiveness to LPS exposure [2]. Therefore, it may be postulated that phenotypic variation in the response to LPS between individuals may be partially controlled by epigenetic modification. Environmental exposures have been linked to alteration in the innate immune response as well, with studies conducted on pregnant rats showing that prenatal exposure to LPS leads to a suppressed innate immune response in offspring when examined at 5 days post birth [3] or even after 40 weeks of life [4]. Understanding the role of DNA methylation on the LPS response as an animal ages, may in time yield candidate regions of control to investigate differential responses to LPS between animals.

We have previously demonstrated an age-dependent increase in the immune response of bovine dermal fibroblasts [5], with cultures from collected the same individual at 16 versus 5 months of age showing an increase in IL-8 production in response to LPS. Understanding the potential epigenetic mechanisms regulating the development of the bovine innate immune response within an individual could be used to help understand underlying causes of variation between individuals. For example, dairy cows display a range of responses when exposed to the same bacterial pathogen in experimental mastitis challenge studies [6,7]. We have also found a substantial range between animals in the magnitude of response of fibroblasts to LPS stimulation that relates to the *in vivo* response to intravenous LPS [5] or intramammary E. coli challenge [8]. The use of fibroblasts collected from the same animal at different ages allows for the investigation of phenotypic variation without confounding genotypic differences.

One potential mechanism controlling the TLR response pathway may be DNA methylation. Some data exists on the role of DNA methylation affecting the TLR4 signaling pathway in humans [1,2], though only limited data exists for dairy cows [9]. In addition, changes in DNA methylation with age have previously been described, further implicating it as a potential mechanism of age associated alterations in gene expression and innate immune response. Analysis of human fibroblasts utilizing the Infiniun HumanMethylation27 Assay, which investigates methylation levels at approximately 27,000 CpG loci, identified both site specific and regional alterations of methylation levels when comparing younger (<23 years old) with older (>65 year old) individuals [10]. In a separate longitudinal study, use of the Infinum HumanMethylation27 Assay found methylation differences between individuals at ages 1 and 5 years based upon hierarchical clustering, denoting changes both within and across individuals due to age [11]. Our work aims to investigate whether a similar phenomenon may be occurring in the bovine model, and whether this may be linked to alterations in cell signaling and subsequent physiological processes.

While the bovine innate immune response has been well characterized under both *in vitro* and *in vivo* conditions [5,12,13], little research has been conducted to determine the development of the response to lipopolysaccharide due to age within an individual. In addition, though a factor with potentially broad implications in gene expression and disease, there is only a limited data set available detailing DNA methylation in the economically important bovine model. Much of the previous work investigating the development of the innate immune response was conducted using mixed cell models from different individuals, which may yield false positive results due to variable cell-mixture proportions rather than true changes in methylation levels [14,15]. This study uses a pure cell model, and eliminated genotypic variation by isolating cells from the same individual at different ages reducing much of the technical and biological variation that may have been present in previous studies offering a clear view of the age-dependent changes in TLR4 signaling.

This study aimed to map transcriptomic alterations in the TLR4 response pathway of bovine dermal fibroblasts isolated from the same individuals at different ages and to determine what effect changes in DNA methylation patterns have on this response. To accomplish this, RNA-Seq was performed on bovine dermal fibroblasts exposed to LPS for 0, 2, and 8 hours. In addition, methylated CpG island recovery assay sequencing (MIRA-Seq) was performed on fibroblast DNA from the same individuals collected at the two different ages. This project allowed for the first in depth look at transcriptome wide changes in gene expression due to age in the bovine fibroblast model while also creating the first descriptive study of the bovine fibroblast methylome and its potential role in altering the innate immune response within an individual over time.

## Results

### Fibroblast challenge with LPS and epigenetic modification

**Figure 1:**
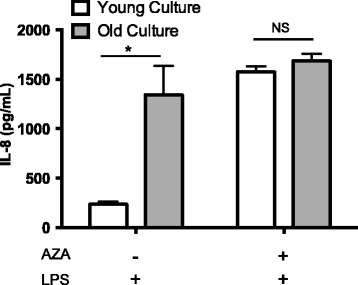
**Fibroblast response to LPS under control conditions or following DNA methylation modification.** Production of IL-8 following exposure to LPS (100 ng/ml) for 24 hours after undergoing pre-treatment with media alone or AZA by bovine dermal fibroblasts. Cultures collected from the same individual at two different ages (young = 5 months of age; old = 16 months of age; n = 3/group) were investigated. *indicates P < 0.05. NS indicates no significance. All values are mean ± S.E.M.

Fibroblasts isolated from 15 dairy heifers at ~5 and 16 months of age have previously been described as showing an age-dependent increase in IL-8 production in response to LPS *in vitro* [5]. To determine if DNA methylation may have contributed to this age effect, pairs of fibroblast cultures collected at 5 and 16 month of age from three mid-level responding animals were selected for epigenetic modification using the demethylating agent AZA prior to LPS exposure for 24 h. Neither control nor AZA treated cultures produced detectable IL-8 in the absence of LPS. However, in response to LPS, young and old cultures displayed greater than 5-fold differences in IL-8 production (P < 0.001), with 5-month-old fibroblasts producing 240 ± 30 pg/ml in contrast to 1,350 ± 290 pg/ml produced by the older cultures (Figure 1). Following AZA treatment, the differential response between age groups was abolished (P > 0.05) with increased IL-8 levels of 1,580 ± 50 pg/ml and 1,690 ± 70 pg/ml produced by the cultures from younger and older individuals, respectively. The cultures from 5 month old animals in particular displayed much higher (P < 0.01) LPS-induced IL-8 production following AZA treatment, while the increase in production from 16-month old cultures, though still significant (P < 0.05), was more muted. The effect of AZA exposure to basal expression levels was not measured, but has previously been reported by us within a similar data set [9].

### RNA-Seq analysis of young and old response to LPS

Total RNA samples from each of the three animals, collected at two ages, and exposed to LPS for 0, 2, and 8 hours were prepared for RNA-Seq analysis. This analysis generated approximately 74 million reads per sample following quality control. Alignment to the UMD v3.1 bovine genome yielded 96% of reads falling within alignment parameters and thus an average of 71 million reads per sample. At hour 0, under our definition of expression (CPM >1 in at least 50% of samples), 18,919 targets were detected forming the core transcriptome of the bovine dermal fibroblast under basal conditions. Following LPS exposure, 18,616 and 19,204 targets were detected after 2 and 8 hours, respectively. In comparison to hour 0, exposure to LPS revealed differential gene expression (FDR < 0.05; CPM > 1; Fold Change ≥ 2) of 617 transcripts at hour 2 (414 up- and 203 down regulated; Additional file 1) and 414 transcripts at hour 8 (260 up- and 154 down-regulated; Additional file 2). Among the genes displaying differential induction due to LPS exposure were those involved in the pro-inflammatory response including *IL-8*, *IL-6*, *CCL20*, *TNF*, and *CXCL2* among others (Table 1). In addition to pro-inflammatory cytokines, the presence of LPS induced many Type-I interferon related genes, including *Myxovirus (MX)-1*, *Ubiquitin-like gene (ISG)-15*, and *interferon induced with helicase C domain (IFIH)-1*, among others. This wide array of cytokines and interferon related genes demonstrate the ability of the dermal fibroblasts to recognize and respond to LPS.

**Table 1:** **Average response to LPS from young and old cultures as measured by increase in expression of immune associated genes as compared to hour 0 post-LPS**

**Gene symbol**	**Gene name**	Fold change*	
		**Hour 2**	**Hour 8**
**Transcription and activation pathways**			
**BIRC3**	Baculoviral IAP repeat-containing protein 3	6.1	4.1
**NFKBIA**	Nuclear factor of kappa light polypeptide gene enhancer in B-cells inhibitor, alpha	36.1	10
**NFKBIZ**	Nuclear factor of kappa-B inhibitor zeta	23.8	4.1
**NFKB2**	Nuclear factor of κ light polypeptide gene enhancer in B-cells 2	2	2.1
**Cytokines and chemokines, growth factors**			
**CCL2**	Chemokine (C-C motif) ligand 2	28.5	26.7
**CCL5**	RANTES	35.4	48.4
**CCL20**	Chemokine (C-C motif) ligand 20	2034	1162.3
**CSF2**	Colony stimulating factor 2	97	6.1
**CXCL1**	Chemokine (C-X-C motif) ligand 1	2.5	- -
**CXCL2**	Chemokine (C-X-C motif) ligand 2	43.8	27.1
**CXCL5**	Chemokine (C-X-C motif) ligand 5	43.8	27.1
**IFNB**	Interferon beta precursor	2	2
**IL1A**	Interleukin 1, alpha	86.0	23.5
**IL-6**	Interleukin 6	83	131.6
**IL-8**	Interleukin 8	123.7	273.7
**SAA3**	Serum Amyloid A 3	505.6	2498.3
**TNF-a**	Tumor Necrosis Factor, alpha	125.2	- -
**TNFSF13B**	Tumor necrosis factor (ligand) superfamily, member 13 (BAFF)	- -	4.6
**Type I IFN-related genes**			
**IFI44**	Similar to Interferon-induced protein 44	- -	15.2
**IFIH1**	Interferon induced with helicase C domain 1	- -	15.1
**ISG15**	ubiquitin-like modifier	6.2	63.2
**MX2**	Myxovirus (influenza virus) resistance 2	- -	121.2
**OAS1**	2′-5′-oligoadenylate synthetase 1	- -	24.6
**OAS2**	2′-5′-oligoadenylate synthetase 2	2.8	26.1
**TNFSF10**	Tumor necrosis factor ligand superfamily member 10 (TRAIL)	- -	15.8

*Data obtained by RNA-Seq and presented as fold induction of the indicated gene at either 2 or 8 hours post-LPS in comparison to expression levels at 0 hours post-LPS. All fold changes shown are FDR < 0.05; FC > 2; and CPM >1. - - indicates FDR > 0.05, FC < 2, or CPM < 1.

**Figure 2:**
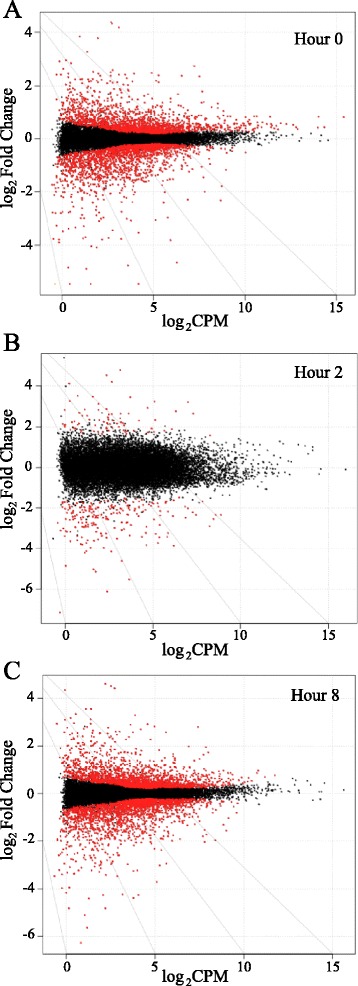
**Scatter plots of RNA-Seq analysis.** Scatter plots of indexes analyzed from RNA-Seq for expression level (log_2_CPM) and differential expression (log_2_ Fold Change) at **(A)** 0 hours, **(B)** 2 hours, **(C)** and 8 hours post-LPS exposure. Positive fold change values indicate higher expression in young cultures while negative values show higher expression in old cultures. Red dots denote FDR < 0.05.

Comparing expression between cultures from younger and older animals revealed 628, 280, and 920 differentially expressed genes (FDR < 0.05; CPM > 1; Fold Change ≥ 2) at 0, 2, and 8 hours post-LPS exposure, respectively (Figure 2; Additional file 3). Of these differentially expressed genes, 400, 206, and 595 displayed higher levels in 16-month old cultures at hours 0, 2, and 8, respectively. The lower number of significantly, differentially expressed genes at hour 2 reflects greater inter-culture variation in response at this time. Pathway enrichment analysis using DAVID indicated that at hour 2 genes within the inflammatory response (P = 6.2 × 10^−9^) and defense response (P = 3.5 × 10^−8^) pathways were the most strongly implicated as different between cultures due to an animal’s age. Similar analysis at hour 8 implicated a larger subset of pathways as being differently expressed, however, inflammatory response (P = 4.3 × 10^−6^) and defense response (P = 5.8 × 10^−7^) were still different between the groups. Among the genes in these pathways displaying differential expression between cultures from younger and older animals at either 2 or 8 hours post LPS were *IL-8*, *IL-6*, *TNF*, and *CCL20* among others (Table 2).

**Table 2:** **Differential expression of immune associated genes at 0, 2, and 8 hours post-LPS exposure of fibroblasts collected at 16 months of age compared to fibroblasts collected at 5 months of age from the same three animals**

**Gene**	**Hour 0**	**Hour 2**	**Hour 8**
**Innate Immune Related Genes**			
CCL2	- -	7.3	3.2
CCL20	- -	18.1	10.1
CCL5	- -	10.7	3.4
CD14	5.5	5.7	4.2
IFIH1	- -	- -	2.1
IL1A	- -	2.3	2.8
IL6	- -	10.5	7.9
IL8	- -	5.8	3.3
MX2	- -	- -	33.9
OAS1	- -	- -	12.8
OAS2	- -	- -	10.6
SAA3	- -	- -	5.1
TLR2	- -	4	2.6
TLR4	2.1	- -	2.6
TNF	- -	10.4	- -
**DNA Methylation Associated Genes**			
DNMT1	1.6	1.6	1.6
DNMT3A	- -	- -	- -
DNMT3B	- -	- -	- -
UHRF1	3.4	3.3	3.6

Data obtained by RNA-Seq and presented as fold change between cultures from younger and older animals. Positive values indicate higher expression in 16-month old cultures as compared to cultures from 5-month old animals. All presented values indicate FDR < 0.05 with an average CPM > 1, while - - notes a values with either FDR > 0.05 or an average CPM < 1.

In addition to genes related to the immune response, those that may be implicated in differential methylation levels were investigated. Among these are the family of *DNMT* genes along with *ubiquitin-like containing PHD and RING finger domains (UHRF)1*. Expression of *DNMT3A* and *3B*, which are involved in *de novo* methylation, showed no differences between cultures collected from younger and older animals. Expression levels of *DNMT1* and *UHRF1* however, involved in DNA methylation maintenance, were significantly higher in 16-month old cultures cultures at all time points. At hour 0, *DNMT1* levels were 1.6 fold higher while *UHRF1* expression was 3.4 fold higher. These levels of differential expression were maintained at hours 2 and 8 post-LPS (Table 2).

### Confirmation of RNA-Seq by RT-PCR

**Figure 3:**
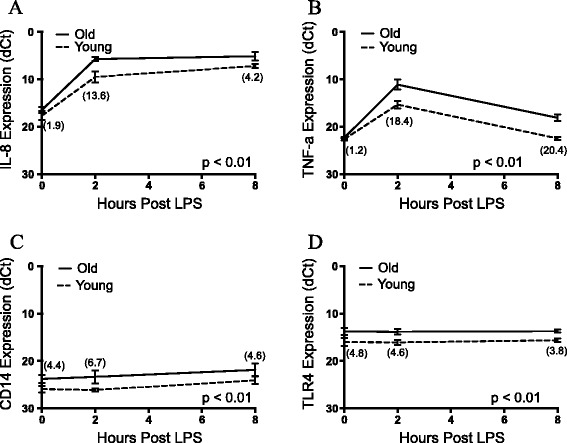
**Comparison of young/old fibroblast response to LPS as measured by RT-PCR.** Comparison of gene expression following exposure to LPS at hours 0, 2, and 8 for young and old cultures. IL-8 **(A)**, TNF-α **(B)**, CD14 **(C)**, and TLR4 **(D)** mRNA were all targeted. Values are expressed as dCt using β-actin expression as the endogenous control. Fold difference in expression between young and old fibroblasts presented for each time point within parentheses. All values are mean ± S.E.M. (n = 3/group). p-values are presented following analysis using a two-way repeated measures ANOVA.

Several genes were selected for RT-PCR confirmation of expression differences between cultures collected from 5 and 16-month old animals that were indicated by RNA-Seq analysis. IL-8, TNF-α, TLR4, and CD14 show consistent values in comparison to those from the RNA-Seq data set. The greatest difference in expression between cultures from the two groups for *IL-8* (Figure 3A) and *TNF-α* (Figure 3B) was at hour 2 post-LPS with 13.6 and 18.4 fold higher expression, respectively, in cultures from 16-month old animals. By hour 8, cultures from older animals were still producing higher levels of both *IL-8* (4.2 fold) and *TNF-α* (20.4 fold), but the differences between the two groups had been reduced. In contrast, *CD14* (Figure 3C) and *TLR4* (Figure 3D) showed little response to LPS, but displayed consistently higher expression in cultures from older animals. For *TLR4*, cultures from 16-month animals displayed 4.8, 4.6, and 3.8 fold higher expression at hours 0, 2, and 8. *CD14* followed a similar pattern, with 4.4, 6.7, and 4.6 fold higher expression in 16-month old cultures at 0, 2, and 8 hours, respectively. *CCL20* (Figure 3E) and *SAA3* (Figure 3F) displayed a strong response to LPS in cultures from both groups, but were more highly expressed in the 16-month old group. No differences in expression were seen at hour 0, however cultures from the older animals produced 17.6 and 32.0 fold higher transcript levels of *CCL20* at hours 2 and 8, respectively. *SAA3* expression was not different between ages at hours 0 or 8, but at hour 2, 16-month old cultures produced 5.6 fold more transcripts. The gene expression values generated by RT-PCR were all in general agreement with RNA-Seq data, validating the transcriptomic results.

### MIRA-Seq

**Figure 4:**
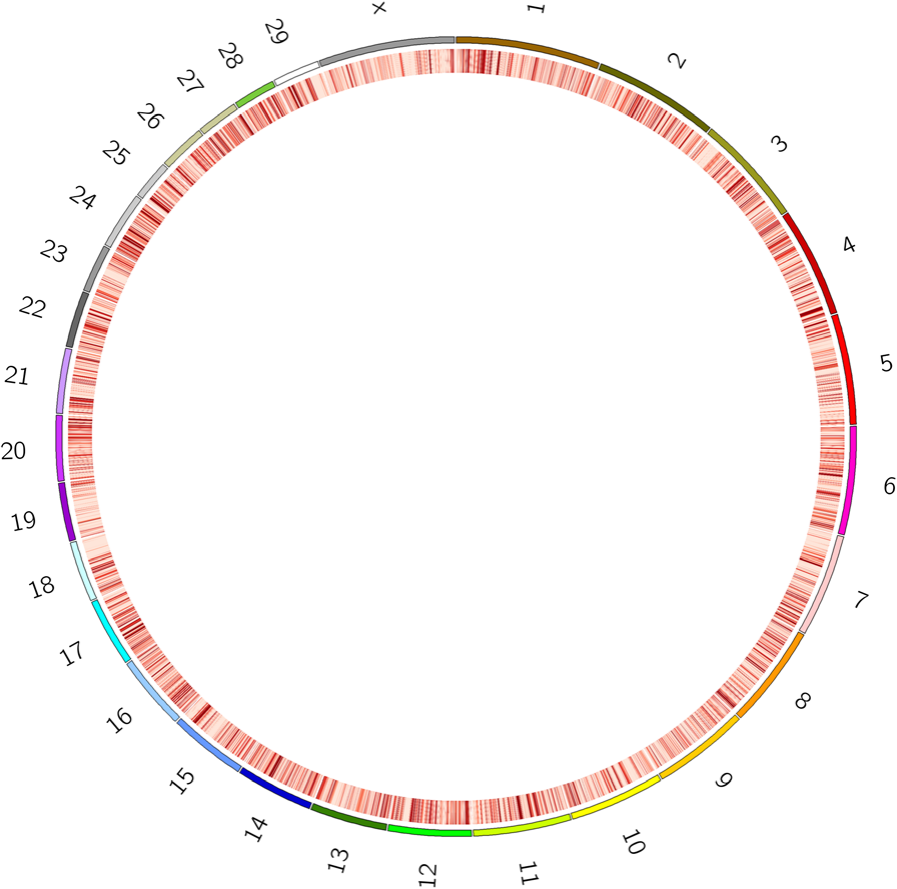
**Circos heat map representing methylation levels across the bovine genome.** Heat map of the bovine fibroblast methylome. Resolution of construction allowed for a single band to represent a 50Kb chunk of the genome. As indicated, darker red represents heavier levels of methylation within that region.

The 6 MIRA-Seq samples resulted in an average of approximately 51 million reads per library following quality control. Alignment to the UMD v3.1 bovine genome yielded 96% of reads falling within alignment parameters and an average of 49 million mapped reads per sample. The genome was analyzed by construction of sequential 3Kb windows in which the read count between cultures from the two age groups were compared. Analysis of the 3Kb genome scan yielded 20 regions that showed significantly different levels of methylation between groups (FDR < 0.1; CPM > 1; FC ≥ 2). Of these differentially methylated regions (DMRs), 13 displayed higher methylation levels in the cultures from 5-month old animals, while 7 indicated higher methylation in cultures from 16-month old animals. Of the 13 DMRs displaying higher methylation levels in 5-month old cultures, 7 were found at least partially within an annotated gene while 3 of the 7 DMRs showing higher methylation in 16-month old cultures were within annotated genes (Table 3). No differences were seen between cultures from the two groups however at the level of promoter methylation, as determined by our definition of −2500 bp to + 500 bp from transcription start site. MIRA-Seq data was also used to construct a heat map of the bovine fibroblast methylome using the Circos software package to illustrate the genome-wide nature of DNA methylation (Figure 4).

**Table 3:** **Differentially methylated 3 Kb regions between young and old individuals as identified by MIRA-Seq and gene expression data for annotated genes**

				**RNA-Seq fold difference**		
Fold difference*	**Chr**	**Genomic coordinates**	Gene**	**Hour 0**	**Hour 2**	**Hour 8**
4.6	1	72956301-72959300	XXYLT1	1.0	−1.6	−1.0
−5.3	2	25773034-25776033	SP5	1.0	1.0	1.0
3.7	2	70978934-70981933	- -	- -	- -	- -
4.7	2	126997334-127000333	ARID1A	1.0	−1.4	1.0
5	2	88469334-88472333	- -	- -	- -	- -
4.9	3	116651410-116654409	CXCR7	−1.2	−1.6	−1.3
4.9	5	117094706-117097705	- -	- -	- -	- -
4	7	52484546-52487545	- -	- -	- -	- -
−4	8	75361987-75364986	ADRA1A	1.0	1.0	1.1
4.2	8	43784287-43787286	DMRT2	−1.1	−1.3	−1.2
6.1	8	101360987-101363986	PALM2	−1.7	−2.4	−1.9
4.7	9	95637251-95640250	- -	- -	- -	- -
−4.3	11	8944985-8947984	LOC100337252	5.2	−1.6	2.5
3.7	11	95333785-95336784	- -	- -	- -	- -
−3.8	12	80725922-80728921	- -	- -	- -	- -
−6.2	17	62367994-62370993	- -	- -	- -	- -
−5.3	17	62346994-62349993	- -	- -	- -	- -
3.6	19	44563675-44566674	HDAC5	1.1	1.2	−1.5
−4.3	21	65927663-65930662	- -	- -	- -	- -
5.9	23	8944593-8947592	ANKS1A	1.3	−1.2	1.1

*Data obtained by MIRA-Seq and presented as fold difference in read count of methylated regions. Positive fold change indicates higher methylation levels in cultures from younger animals while negative values are higher methylation in cultures from older animals.

**DMRs with an associated gene indicate that some portion of the 3Kb region falls within an annotated gene, while - - indicates DMRs that are intergenic.

### The role of DNA methylation on gene expression

**Figure 5:**
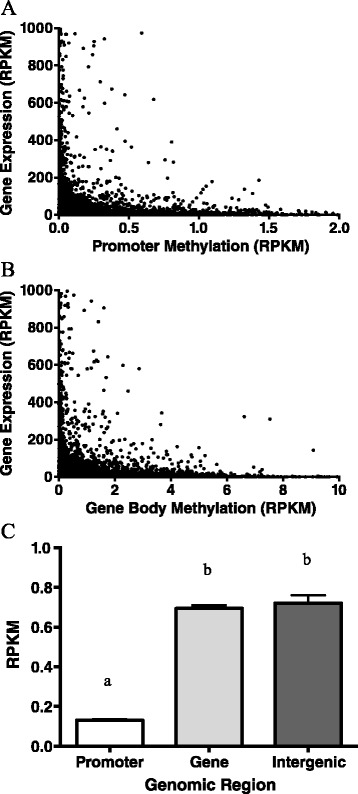
**The role of DNA methylation on gene expression and differential methylation levels due to genomic region.** Scatter plot showing gene expression at 8 hours post-LPS exposure in relation to the level of DNA methylation at the gene’s **(A)** promoter or **(B)** within the gene itself. Expression levels for RNA-Seq and methylation data were normalized to gene size and presented as RPKM. Each value represents the average expression of the six young and old cultures investigated. Data obtained by RNA-Seq and MIRA-Seq. **(C)** Average methylation levels based upon genomic region for young/old MIRA-Seq libraries. Lettering denotes differential levels of methylation as measured by a one-way ANOVA with Bonferonni post-test (p < 0.05). All values are mean ± S.E.M.

The association between methylation levels within a gene body or gene promoter region and that gene’s levels of expression was analyzed by Fisher’s exact test. MIRA-Seq values for a gene body or gene promoter were plotted against RNA-Seq expression levels at 8 h post-LPS. Each data point was binned as either being a high or low, with a gating value of reads per kilobase per million matched reads (RPKM) =5 or RPKM = 0.5 for RNA-Seq and MIRA-Seq, respectively. Analysis of the relationship between promoter methylation level and that gene’s subsequent expression level showed that the two values were significantly (P < 0.001; O.R. = 1.53; 95% C.I. = 1.39-1.67) dependent upon one another, and indicating a strong inverse relationship between gene promoter DNA methylation and gene expression (Figure 5A). The relationship between gene body methylation levels and that gene’s expression levels were also significantly (P < 0.05; O.R. = 1.15; 95% C.I. = 1.05-1.26) dependent upon one another, though to a smaller extent than promoter methylation (Figure 5B). The 8-hour time point was selected for presentation as this offered the period with the greatest number of genes showing high expression levels as analyzed by edgeR. Analysis of the 0 and 2-hour post-LPS times gave similar results (data not shown).

DNA methylation levels were also measured in relation to genomic region. Methylation was measured as RPKM in gene promoters, gene bodies, or intergenic regions and revealed that promoter regions have significantly less methylation than gene body or intergenic regions while these two showed no difference from one another (Figure 5C).

## Discussion

The ability of an individual to recognize and respond to bacterial components such as LPS during an infection plays a critical role in host defense [16]. Previous work has suggested that neonates are deficient in innate immune responsiveness in comparison to adults [17,18] however these studies utilized mixed-cell models and were subject to highly variable responses. In using bovine fibroblast cultures, we are able to examine a single population of cells isolated from the same animal at different times, but cryopreserved and once revived, exposed to LPS in side by side wells, eliminating much of the technical and genetic variability seen in these previous studies. A broader understanding of the regulated development of the innate immune response within an animal would potentially allow the application of this knowledge to determining factors that may be producing variability between animals in their response to different pathogens.

The goals of this study were to further our previous observation of age-dependent differential gene expression in response to LPS, and to determine what role DNA methylation may be playing in this process. Previously we had found that fibroblast cultures from the same individual but collected at either five or sixteen months of age showed distinct differences in their ability to produce IL-8 in response to LPS, with the cultures from older animals displaying a more hyper-responsive phenotype [5]. In addition, we found that variability in the response between individuals could be abolished with the exposure of cultures to AZA, a global demethylating agent, thus implicating DNA-methylation as a factor contributing to the variation [9]. In the current experiment, we expand on these results and explored the bovine transcriptome to fully describe age-dependent differences in gene expression following LPS exposure.

### Differential response to LPS due to age

RNA-Seq data showed a great deal of differential expression between cultures from younger and older animals, predominantly cytokine and chemokines previously shown to play an important role in the resolution of bacterial infections such as *E. coli* mastitis [8,19]. In addition, TLR4, the extracellular receptor of LPS, as well as CD14, an important co-receptor in the TLR4 signaling pathway [20] were differentially expressed. The identification of LPS recognition genes suggests that cultures from older animals are more readily able to detect danger signals by the binding of PAMPs. Subunits of the NF-κB complex, namely p50 and p52 showed higher expression (1.4 fold in both) in 16-month old cultures 8 hours post-LPS exposure, but were not differentially expressed due to age at 0 or 2 hours post-LPS.

Another cytokine of interest is IL-1α, which can have a strong autocrine effect on cells producing it [21]. Of interest is the fact that cultures from older animals produced 2.3 and 2.8 fold higher levels than cultures from young animals at 2 and 8 hours post-LPS respectively. Intracellular IL-1α has been linked to increased sensitivity to inflammatory stimuli by NF-κB and AP1 suggesting that it plays an important role in the modulation of the inflammatory response [22]. This correlates with related research showing responsiveness to LPS exposure in airway epithelia of rhesus monkeys, as measured by IL-8 protein production as well as IL-1α transcript, increased in an age dependent manner [15]. Research on the response to LPS in human monocytes and dendritic cells confirmed an increase in the TLR signaling response with age as responsiveness of infants increased over time reaching adult levels within one year of age [18]. The gene expression data presented here, along with the work conducted by others suggests that individuals undergo a distinct maturation of response to LPS.

### Temporal response to LPS

The RNA-Seq data also presents a comprehensive view of the temporal aspects of the LPS response. There are several hundred genes displaying age-dependent differential expression at hour 0, presenting a picture of the basal variation in expression within an individual due to age. After 2 h of LPS exposure there is a marked reduction in the absolute number of differentially expressed genes due to high levels of variability across the cultures sampled. This would appear to indicate that within the first two hours of LPS exposure, the machinery controlling gene expression is in flux limiting discernable bias between young and old cultures in the speed in which they responded. However, by 8 hours post-LPS, variability within age groups had been reduced and a greater number of significant differences are seen between the cultures. A similar look at the effects of time on the transcriptome of bovine mammary epithelial cells in response to LPS showed low levels of differential expression due to treatment at an early time point (3 h) in comparison to a later time (6 h), demonstrating that the early response to LPS may be more affected by rapid changes in gene expression making it difficult to generate statistical significance between groups [23].

### Expression of DNA methylation-associated genes

RNA-Seq analysis also highlighted differences in genes involved in the maintenance of DNA methylation. Cultures from older animals showed higher levels of expression of *DNMT1* the catalytic methyltransferase enzyme responsible for the addition of the methyl group onto an unmethylated cytosine residue [24]. Expression levels were also elevated in 16-month old cultures cultures for *UHRF1*. This gene product identifies hemi-methylated DNA following mitosis, recruits DNMT1 to the site, and physically flips the methylcytosine residue from the parent strand out of the double helix to sterically allow access of DNMT1 to the demethylated daughter strand [25,26]. The lower levels of *DNMT1* and *UHRF1* expression in the cultures from younger animals potentially suggest these cultures may be less engaged in high fidelity maintenance of methylation levels. In contrast, cultures from older animals, having gone through a major developmental shift, are now expressing higher levels of both genes in an effort to maintain DNA methylation. However, differences in DNA methylation due to differential expression of *DNMT1* and *UHRF1* would potentially occur as minor changes rather than active, wholesale modifications in DNA methylation status.

### The role of methylation in the development of the innate immune response is limited

The innate immune response in intestinal epithelial cultures had previously been linked to DNA methylation of the NF-κB inhibitor IκBα as measured by production of IL-8 following TNF-α exposure [27]. In our experiments the relative increase in LPS-induced IL-8 response following AZA treatment was much greater in fibroblasts from younger individuals. This eliminated the differences seen between the cultures due to age in the absence of AZA, suggesting that DNA methylation plays an important role in the development of the immune response. To more directly determine where DNA methylation was changing over time within individuals we performed MIRA-Seq on DNA from fibroblast cultures established from skin biopsies collected from three animals at two ages.

While RNA-Seq data showed major changes in the transcriptome of an individual due to age, MIRA-Seq analysis of the bovine methylome showed fewer alterations than expected. Our methylation analysis identified only twenty 3Kb regions that were differentially methylated between younger and older individuals. While our comparison within individuals showed little statistically significant changes in methylation levels, this was not due to high variability between individuals. This would appear to indicate that there is a limited change in methylation seen within an individual over the 11-month period investigated within this study. While highly variable methylation levels have been reported in healthy human populations there has been a more limited description of methylation differences within individuals [28,29]. The results from our data suggest that there are a restricted number of wholesale age-dependent changes in the fibroblast methylome that can be detected by the MIRA-Seq analysis. However in light of the results following AZA exposure it appears that more subtle changes, not detectable by MIRA-Seq, are occurring that affect the innate immune response. DNA methylation quantification by MIRA-Seq has been shown to be less sensitive due to the nature of the assay in comparison to other methods, including reduced representation bisulfite sequencing and whole genome bisulfite sequencing, which would limit the ability of our study to detect any minor changes in methylation levels [30].

A limitation of the current study is the low-resolution nature of MIRA-Seq. Exposure of cultures to AZA demonstrated that DNA methylation is playing a role in the development in the immune response by rescuing the phenotype of young cultures. While MIRA-Seq allows for an overall visualization of the methylome and can discern differences in overall quantity of methylated CpGs down to approximately a 100 bp resolution, subtle methylation patterns are missed. This is problematic as regions altered by just a few CpG dinucleotides have been shown to have appreciable effects on gene expression [31,32]. Previous studies that successfully found differences in methylation due to development did so with a single base pair resolution [33,34]. In addition, work conducted in human epidermal tissues found that the overall methylome was relatively stable within an individual over time, and alterations were limited to minor, local changes which again would be lost in our analysis [33]. Future studies would benefit from an increase in the resolution of the analysis technique.

### The role of methylation on bovine fibroblast gene expression

MIRA-Seq data allowed for the construction of a genome-wide DNA methylation heat map. This map, the first built for the bovine fibroblast methylome, displayed dense regions of methylation interspersed throughout all of the genome. As a composite of the six MIRA-Seq libraries created, we are able to see regions of methylation likely playing major regulatory roles in fibroblast gene expression and differentiation. While there is currently a dearth of information on the methylation status of different bovines tissues, the construction of the fibroblast methylome is an important first step towards a comparative analysis of methylation across cell types within the bovine system.

MIRA-Seq analysis of our samples was able to confirm several important phenomena seen previously in other target species in terms of DNA methylation’s relationship to gene expression, and the non-random placement of methylated CpG dinucleotides across the genome. Our data reaffirmed the relationship between gene expression and methylation levels in that heavily methylated genes had a significant tendency towards lower expression levels [35]. In addition, our data suggested that the placement of methylation along DNA is not a random process, but biased in relation to DNA sequences with distinct functions. More specifically, methylation levels are clearly depleted in the promoter region of genes. Analysis also showed no differences in methylation levels between gene and intergenic regions, suggesting that the promoter region of a gene is highly sensitive to methylation and the one most highly regulated in terms of *de novo* DNA methylation, confirming what has been previously reported [33].

While RNA-Seq technology was able to define major differences in the recognition and response of bovine dermal fibroblasts to LPS, data investigating the role of DNA methylation in this process was inconclusive. The results from this investigation would suggest that major regulatory changes are occurring within the fibroblasts of individuals due to age, potentially due to differential levels of methylation, or a change in the sensitivity to DNA methylation due to age. As these genes have demonstrated the ability to be regulated due to age alone, it is possible to determine biomarkers from this study to identify malleable candidate genes that can potentially be manipulated to produce a more ideal phenotype. While our results focused on the innate immune response, it would seem likely that many cellular processes are undergoing similar alterations during development. In addition, our data using AZA as a global de-methylation agent points to DNA methylation as a major player in the alteration of cellular responsiveness to LPS. A MIRA-Seq-based genome-wide scan of methylation however was able to find only a handful of candidate regions differentially methylated due to age. These regions require further investigation with a more sensitive tool for measuring methylation levels on a larger sample size.

## Conclusions

The LPS-induced transcriptome of bovine dermal fibroblasts is greatly altered due to age, with epigenetic regulation playing a potentially important role. Using multiple cultures from single individual would appear to give researchers an ideal model for investigating not only the role of aging in disease but also general mechanisms controlling the innate immune response. By investigating effectors altering LPS responsiveness, this data may be applicable to finding variations within a population in relation to variation in phenotypic response to pathogens.

## Methods

### Animals and experimental design

All experiments were performed in accordance with approval of the Institutional Animal Care and Use Committee at the University of Vermont. Three pairs of dermal fibroblast cultures, collected from the same animals at 5 and 16 month of age, were selected from a previously described collection of cultures obtained from 15 Holstein heifers [27]. Fibroblast isolation from the 15 animals and their ability to produce IL-8 in response to LPS has previously been described [5]. The selected culture pairs for the current experiment came from three mid-level responding animals. Mid-level cultures were selected in order to not artificially increase variation within the investigated population as to ensure that differences seen due to age were not lost to inter-animal variation. The rankings of these cultures remained consistent across samplings and age had no effect on relative response [5]. Sampling at each age occurred within a several week timeframe, though no batch effect was detected to play a role in methylation level or LPS response (data not shown). To test the presented hypothesis, three distinct experiments were performed: IL-8 protein analysis, RNA-Seq, and MIRA-Seq. Within each experiment, LPS challenge or RNA/DNA collection were conducted at the same time in side-by-side wells. Each experiment utilized a different aliquot of cryopreserved cells from the individuals examined, though the aliquots were all taken from 5th passage cells to control for cell expansion dynamics, including any potential alterations in DNA methylation levels due to growth.

### Fibroblast epigenetic treatment and LPS challenge

Following recovery from cryopreservation and expansion in a T-75 cm^2^ flask, cells were trypsinized and seeded in 6-well culture plates at 1.25 × 10^5^ cells/mL in a total volume of 2 mL. Growth media consisted of DMEM with 5% fetal bovine serum (Thermo-Scientific, Waltham, MA), 1% insulin-transferrin-selenium (Life Technologies, Carlsbad, CA), and 1× penicillin/streptomycin (Thermo-Scientific) at 37°C with 5% CO_2_. Cells were exposed to 10 μM 5-aza-2′deoxycytidine (AZA; Sigma, St. Louis, MO) for 96 hours to achieve DNA demethylation, which constituted our epigenetic modification treatment. Control cells were grown for 4 days with comparable amounts of the AZA vehicle (PBS) added. Dosages and exposure time to AZA were modeled after previous experiments conducted that displayed low cytotoxicity from treatment in conjunction with effective epigenetic remodeling at similar or lower concentrations than those used in our trials [1,36,37]. Cell viability measurements were not conducted for each individual AZA exposure presented here, however, previous trials utilizing AZA modification demonstrated >99% viability for all cells examined across multiple replicates.

Following epigenetic modification, cells were washed 3× with PBS and exposed to LPS for 24 hours. LPS treatment consisted of growth media supplemented with 100 ng/mL of ultra-pure LPS isolated from *Escherichia coli* O111.B4 (Sigma-Aldrich). Following appropriate exposure time, media was removed, spun at 500 × g for 5 minutes to remove cell debris, and immediately stored at −20°C until further analysis.

### Quantification of IL-8

The concentration of IL-8 in fibroblast conditioned media was quantified by a custom sandwich ELISA previously described [8] using mouse anti-bovine IL-8 monoclonal antibody (clone 170.13 generously gifted by S. Maheswaren, University of Minnesota) and biotinylated goat anti-human IL-8 antibody (R&D Systems Inc., Minneapolis, MN) as capture and detection antibodies, respectively, and recombinant bovine IL-8 (Thermo Scientific, Rockford, IL) as assay standard. The detection limit of the assay was approximately 300 pg/ml. IL-8 protein production was analyzed between groups (cultures from younger vs. older animals) using a paired Student’s t-test (Graph Pad Prism 6.0). Analysis of real time gene expression data was conducted using a two-way ANOVA model with repeated measures (Graph Pad Prism 6.0). Comparisons with *P* < 0.05 were considered statistically significant within experiments.

### RNA-Seq

Additional aliquots of the fibroblast cultures that were collected at 5 and 16 months of age from the same three animals were revived from cryopreservation, expanded, and seeded in duplicate wells of a 6-well culture plates at 1.25 × 10^5^ cells/mL in a total volume of 2 mL. After two days the cultures were exposed to LPS (100 ng/ml) 0, 2, and 8 hours in the presence of LPS. Total mRNA was collected using the 5-Prime PurePerfect RNA Purification Kit (Gaithersburg, MD), which includes a DNase treatment step to eliminate DNA contamination. Resulting RNA was quantified and assessed using a Qubit Spectrofluorometer (Life Technologies Carlsbad, CA) and an Agilent Bioanalyzer 2100 (Agilent technologies Santa Clara, Ca) to ensure that all samples had an RNA integrity number (RIN) value of 8 or greater. Library construction was carried out using Illumina TruSeq RNA Sample Prep LT version 2 (Illumina Inc. Boston, MA). Briefly, 500 ng of total RNA was PolyA enriched using magnetic beads followed by reverse transcription using Superscript II (Invitrogen). cDNA was then fragmented, end repaired, and adenylated followed by a ligation of Illumina adaptors with a unique adaptor sequence (barcode) for each sample. PCR amplification was performed using Illumina Reagents followed by quantification and assessment of quality (as described above) along with high accuracy qPCR quantitation (KAPA Biosciences kit # 4824; Barre, VT). All DNA clean-up steps were performed with the AMPure XP Magnetic Beads (Beckman Coulter). Sequencing was performed using 12pM/flow cell lane on an Illumina CBOT for flow cell cluster generation and the HiSeq1000 for sequencing by Synthesis equipped with the HiSeq Control and sequence Analysis Software. The 18 samples were multiplexed across 7 lanes of a flow cell and sequenced as a single end 100 bp read.

Raw sequence reads were filtered to eliminate reads that had a median quality (Q) score below 20, more than 3 uncalled bases, or were less than 25 bp following trimming and aligned to the reference UMD v 3.1 bovine genome using the software package NextGENe v. 2.3.4 (Softgenetics, State College, PA). Alignment parameters required >85% of the each read’s length to map to the reference sequence for it to be considered a mapped read. After reads were mapped with NextGENe, total raw read counts for each gene were generated. These read counts were used for further statistical analysis to determine differentially expressed genes as described below. RNA sequencing data is publicly available within the gene expression omnibus (GEO) repository as GSE61027 within the SuperSeries GSE61168.

### DNA isolation and methylated CpG Island recovery assay (MIRA-Seq)

MIRA-Seq libraries were created to investigate genome wide methylation levels in fibroblasts from the 3 individuals at 5 and 16 months of age (n = 3/age). 1.5 ug of genomic DNA was isolated from fibroblasts having no previous LPS exposure using the 5-Prime (Gaithersburg, MD) Pure Perfect Archive DNA Extraction kit and sonicated in 150 ul of water for 10 minutes with alternating cycles of 30s on followed by 30s off using a Diagenode Bioruptor 300 (Denville, NJ). Sonicated DNA was purified using the Qiagen MinElute PCR Purification kit. Fragmented DNA then underwent end repair using New England Biolabs (Ipswich, MA) NEBNext End Repair Module following manufacturer’s instructions. End-repaired DNA was recovered with AMPure XP Beads (Beckman Coulter, Pasadena, CA) per manufacturer’s instructions and then dA-tailed using the New England Biolab NEBNext dA-Tailing Module. Following subsequent cleanup using the AMPure XP Beads, universal adapters were ligated onto the DNA fragments using T4 DNA ligase (NEB). Barcoded adaptors were then attached with the NEBNext Multiplex Oligos for Illumina kit (NEB) followed by purification using AMPure XP Beads.

Next, MIRA pulldown was performed using the Methyl Collector Ultra Kit (Active Motif, Carlsbad CA) per manufacturer’s instructions. The methylated DNA rich library DNA was then run out on a 1% agarose gel with 1% SYBR Green and fragments of approximately 300-700 bp in size were excised. DNA was then eluted from the gel using the Qiagen MinElute Gel Extraction kit.

PCR amplification of the libraries was conducted using the NEBNext DNA Library kit universal adaptors with Amplitaq Gold with GeneAmp (Life Technologies, Carlsbad, CA). Cycling conditions were: initial denaturation at 98°C for 30 seconds; then 14 cycles consisting of denaturation at 98°C for 10 seconds, annealing at 65°C for 30 seconds and extension at 72°C for 30 seconds with a final extension of 72°C for 5 minutes. PCR products were cleaned using the AMPure XP Beads and run on a 1% agarose SYBR Green gel to remove excess primers as previously described. The DNA library was excised from the gel then purified using the Qiagen MinElute Gel Extraction kit. Each library was tested for enrichment of methylated DNA by performing PCR using a primer pair designed to amplify a known methylated gene (LIT1) as a positive control as well as a primer pair that amplifies an unmethylated gene (MP68) to ensure adequate depletion of unmethylated DNA. LIT1 primer sequences were 5′-TGCTCTGGACGTGGTCCGCCTGG-3′ for forward sequence and 5′-CCGGGCGACCGTGGCGACCT-3′ for reverse. MP68 sequences were 5′-GGGCCTGGGCCTTGCCCTCA-3′ for forward and 5′-TGCAAAAGGCTAGCTGGCTGCAAT-3′ for reverse. Sequencing of the libraries was performed using a 12pM/lane bridge amplification on an Illumina CBOT for flow cell cluster generation and the HiSeq1000 for sequencing by Synthesis equipped with the HiSeq Control and sequence Analysis Software. MIRA sequencing data is publicly available within the GEO repository as GSE61101 within the SuperSeries GSE61168.

### Analysis of RNA-Seq

Raw sequence reads were filtered to eliminate reads that had a median quality (Q) score below 20, more than 3 uncalled bases, or were less than 25 bp following trimming and aligned to the reference UMD v 3.1 bovine genome using the software package NextGENe v. 2.3.4 (Softgenetics, State College, PA). Alignment parameters required >85% of the each read’s length to map to the reference sequence for it to be considered a mapped read. After reads were mapped with NextGENe, total raw read counts for each gene, as defined by the UCSC build, were generated.

RNA-Seq data was analyzed by the well-established statistical methods employed by edgeR in the R software package (version 3.0.1). As an initial step, genes with low read counts, defined as at least one mapped read per million mapped reads (counts per million; CPM) in less than 50% of the samples being compared, were eliminated. For example, comparison of cultures from younger and older animals at a given time point employed analysis of n = 3 samples/group, so at least 3 samples needed a CPM equal to or greater than 1 to be considered for analysis.

Comparison of cultures from the animals at different ages at different time points for RNA-Seq was conducted using a paired generalized linear model likelihood-ratio test using the limma package. Paired analysis was also used to determine the response to LPS (0 h vs. 2 h, and 0 h vs. 8 h post-LPS). Raw p-values were adjusted to account for multiple comparisons using the Benjamini-Hochberg method [38].

When analyzing the effects of age on the LPS response, genes were considered differentially expressed if they passed the false discovery rate (FDR) < 0.05 and fold-change (FC) ≥ 2 thresholds. To determine the effects of LPS on gene expression, cultures at the 2 and 8-hour time points were compared to 0 h cultures. The Database for Annotation, Visualization and Integrated Discovery (DAVID; http://david.abcc.ncifcrf.gov) was used for functional annotation and analysis by uploading the official gene symbol of statistically significant genes (FC ≥ 2; CPM > 1; FDR < 0.05 for RNA-Seq).

### Analysis of MIRA-Seq

Raw sequence reads were filtered to eliminate reads that had a median quality (Q) score below 20, more than 3 uncalled bases, or were less than 25 bp following trimming and aligned to the reference UMD v 3.1 bovine genome using the software package NextGENe v. 2.3.4. Alignment parameters required >85% of the each read’s length to map to the reference sequence for it to be considered a mapped read. After reads were mapped with NextGENe, total raw read counts for each gene, as defined by the UCSC build, were generated.

Data was analyzed by in the R software package (version 3.0.1) using the edgeR module. As an initial step, genes with low read counts, defined as at least one mapped read per million mapped reads (counts per million; CPM) in less than 50% of the samples being compared, were eliminated. Comparison of methylation levels for cultures from young and old animals was conducted using a paired generalized linear model likelihood-ratio test using the limma package.

DMR identification was performed on 3Kb windows to allow for an overall scan of the bovine fibroblast methylome. Raw p-values were adjusted to account for multiple comparisons using the Benjamini-Hochberg method [38]. When analyzing differential DNA methylation levels, MIRA-Seq data was considered different between group if they passed the FDR < 0.1 and FC ≥ 2 thresholds. Finally, genome wide methylation was visualized as a composite of the six MIRA-Seq libraries created using the free software package, Circos [39].

### Quantitative real-time PCR

A subset of genes identified by RNA-Seq analysis was selected for expression analysis by quantitative real-time PCR (RT-PCR) using oligonucleotide primers specific to *toll-like receptor (TLR)-4*, *IL-8*, *tumor necrosis factor (TNF)-α*, and *cluster of differentiation (CD)-14* (Table 4). The same RNA samples from the genome-wide expression study were used. First-strand cDNA synthesis was conducted using the Improm-II Reverse Transcriptase Kit (Promega). Messenger RNA expression was quantified by RT-PCR with a CFX96 Real-Time Instrument (Bio-Rad, Hercules, CA) using PerfeCTa SYBR Green Super-Mix, Low ROX kit (Quanta Biosciences). Cycling conditions were: initial denaturation at 95°C for 2 minutes; then 40 cycles consisting of denaturation at 95°C for 15 seconds, annealing at 60°C for 30 seconds and extension at 72°C for 1 minute. All samples were run in duplicate. Melt curve analysis was also performed to check amplification of the desired gene product. The *β-actin* gene was used as reference gene for normalization procedure. Cycles to threshold (Ct) were calculated for each sample and analyzed using the ΔCt method with fold change being 2^**ΔΔCt**^.

**Table 4:** **Primer pairs used for amplification of target genes by RT-PCR**

**Gene**	**Forward primer sequence**	**Reverse primer sequence**	**Reference**
CD14	CTCCAGCACCAAAATGAC	TCCTCTTCCCTCTCTTCC	[40]
IL-8	GCTGGCTGTTGCTCTCTTG	AGGTGTGGAATGTGTTTTTATGC	[41]
TNF	TCTTCTCAAGCCTCAAGTAACAAGC	CCATGAGGGCATTGGCATAC	[42]
TLR4	ACTGCAGCTTCAACCGTATC	TAAAGGCTCTGCACACATCA	[13]
B-Actin	GCAAATGCTTCTAGGCGGACT	CAATCTCATCTCGTTTTCTGCG	[41]

### Analysis of RNA-Seq and MIRA-Seq relationship

In order to determine the relationship between DNA methylation and gene transcription levels, the average reads per kilobase per million matched reads (RPKM) from the RNA-Seq and MIRA-Seq of the six cultures from younger and older animals was investigated. Values were calculated for promoter methylation levels as well as for annotated genes (as defined by the UMD v3.1 genome) from both the MIRA-Seq data and the RNA-Seq data at 0, 2, and 8 hours post-LPS exposure. The relationship of mRNA transcription levels and DNA methylation was then determined by a two-tailed Fisher’s exact test in the R software package in which high or low methylation levels were investigated for an association to either high or low RNA expression levels. For gene expression RPKM values, length was calculated as the cumulative size of the gene exons, while for gene methylation, gene body length was the total size of both intronic and exonic segments. All values were normalized to library and transcript size by conversion of read counts into RPKM values. RNA-Seq RPKM values were binned into either high or low levels at a cutoff of RPKM = 5 while MIRA-Seq RPKM values were divided into high or low levels at RPKM = 0.5.

To determine whether the type genomic region assessed had an affect on DNA methylation levels, average RPKM was calculated for gene promoters, gene bodies, and intergenic regions. Gene body and intergenic regions were determined by annotation from the UMD version 3.1 bovine genome, while gene promoters were defined as −2500 to +500 bp of a gene transcription start site. To determine differential methylation levels based upon genomic location, a one-way ANOVA with a Bonferroni post-test for multiple comparisons was run.

### Availability of supporting data

The data discussed in this publication have been deposited in NCBI’s Gene Expression Omnibus and are accessible through GEO SuperSeries accession number GSE61168 (https://www.ncbi.nlm.nih.gov/geo/query/acc.cgi?acc=GSE61168).

## Additional files

Additional file 1:
**Genes displaying differential expression (FDR < 0.05; CPM > 1; 2 < FC < −2) due to LPS at hour 2 as compared to hour 0. A positive fold change indicates higher expression at hour 2 than at hour 0.** CPM = Counts per Million. FDR = False discovery rate. Data shown for comparisons with FDR < 0.05; CPM > 1, and fold change > 2. Additional file 2:
**Genes displaying differential expression (FDR < 0.05; CPM > 1; 2 < FC < −2) due to LPS at hour 8 as compared to hour 0.** A positive fold change indicates higher expression at hour 8 than at hour 0. CPM = Counts per Million. FDR = False discovery rate. Data shown for comparisons with FDR < 0.05; CPM > 1, and fold change > 2. Additional file 3:
**Differentially expressed genes (FDR < 0.05; CPM > 1; 2 < FC < −2) between young (5 months) and old (16 months) fibroblast cultures from the same individual exposed to 100 ng/ml LPS for 0, 2, or 8 hours.** A positive fold change indicates higher expression in old cultures. CPM = Counts per Million. FDR = False discovery rate.

## References

[1] Takahashi K, Sugi Y, Hosono A, Kaminogawa S (2009). Epigenetic regulation of TLR4 gene expression in intestinal epithelial cells for the maintenance of intestinal homeostasis. J Immunol.

[2] Vamadevan AS, Fukata M, Arnold ET, Thomas LS, Hsu D, Abreu MT (2010). Regulation of toll-like receptor 4-associated MD-2 in intestinal epithelial cells: a comprehensive analysis. Innate Immun.

[3] Hodyl NA, Krivanek KM, Clifton VL, Hodgson DM (2008). Innate immune dysfunction in the neonatal rat following prenatal endotoxin exposure. J Neuroimmunol.

[4] Williams C, Teeling J, Perry V, Fleming T. Mouse maternal systemic inflammation at the zygote stage causes blunted cytokine responsiveness in lipopolysaccharide-challenged adult offspring. BMC Biology. 2011;49(9). 10.1186/1741-7007-9-49 PMC3152940 21771319

[5] Green BB, Kandasamy S, Elsasser TH, Kerr DE (2011). The use of dermal fibroblasts as a predictive tool of the toll-like receptor 4 response pathway and its development in Holstein heifers. J Dairy Sci.

[6] Kornalijnslijper JE, Daemen AJJM, van Werven T, Niewold TA, Rutten VPMG, Noordhuizen-Stassen EN (2004). Bacterial growth during the early phase of infection determines the severity of experimental Escherichia coli mastitis in dairy cows. Vet Microbiol.

[7] Buitenhuis B, Rontved C, Edwards S, Ingvartsen K, Sorensen P (2011). In depth analysis of genes and pathways of the mammary gland involved in the pathogenesis of bovine Escherichia coli-mastitis. BMC Genomics.

[8] Kandasamy S, Green BB, Benjamin AL, Kerr DE (2011). Between-cow variation in dermal fibroblast response to lipopolysaccharide reflected in resolution of inflammation during Escherichia coli mastitis. J Dairy Sci.

[9] Green BB, Kerr DE (2014). Epigenetic contribution to individual variation in response to lipopolysaccharide in bovine dermal fibroblasts. Vet Immunol Immunopathol.

[10] Koch C, Suschek C, Lin Q, Bork S, Goergens M, Joussen S (2011). Specific age-associated DNA methylation changes in human dermal fibroblasts. PLoS One.

[11] Martino DJ, Tulic MK, Gordon L, Hodder M, Richman TR, Metcalfe J (2011). Evidence for age-related and individual-specific changes in DNA methylation profile of mononuclear cells during early immune development in humans. Epigenetics.

[12] Rinaldi M, Li R, Bannerman D, Daniels K, Evock-Clover C, Silva M (2010). A sentinel function for teat tissues in dairy cows: dominant innate immune response elements define early response to E. coli mastitis. Funct Integr Genomics.

[13] Ibeagha-Awemu EM, Lee J-W, Ibeagha AE, Bannerman DD, Paape MJ, Zhao X (2008). Bacterial lipopolysaccharide induces increased expression of toll-like receptor (TLR) 4 and downstream TLR signaling molecules in bovine mammary epithelial cells. Vet Res.

[14] Liao S-L, Yeh K-W, Lai S-H, Lee W-I, Huang J-L (2013). Maturation of toll-like receptor 1–4 responsiveness during early life. Early Hum Dev.

[15] Maniar-Hew K, Clay CC, Postlethwait EM, Evans MJ, Fontaine JH, Miller LA (2013). Innate immune response to LPS in airway epithelium is dependent on chronological age and antecedent exposures. Am J Respir Cell Mol Biol.

[16] Beutler B, Hoebe K, Du X, Ulevitch RJ (2003). How we detect microbes and respond to them: the toll-like receptors and their transducers. J Leukoc Biol.

[17] Kollmann TR, Crabtree J, Rein-Weston A, Blimkie D, Thommai F, Wang XY (2009). Neonatal innate TLR-mediated responses are distinct from those of adults. J Immunol.

[18] Nguyen M, Leuridan E, Zhang T, De Wit D, Willems F, Van Damme P (2010). Acquisition of adult-like TLR4 and TLR9 responses during the first year of life. PLoS One.

[19] Burvenich C, Van Merris V, Mehrzad J, Diez-Fraile A, Duchateau L (2003). Severity of E. coli mastitis is mainly determined by cow factors. Vet Res.

[20] Jiang Z, Georgel P, Du X, Shamel L, Sovath S, Mudd S (2005). CD14 is required for MyD88-independent LPS signaling. Nat Immunol.

[21] Günther J, Esch K, Poschadel N, Petzl W, Zerbe H, Mitterhuemer S (2011). Comparative kinetics of escherichia coli- and staphylococcus aureus-specific activation of key immune pathways in mammary epithelial cells demonstrates that S. Aureus elicits a delayed response dominated by interleukin-6 (IL-6) but not by IL-1A or tumor necrosis factor alpha. Infect Immun.

[22] Werman A, Werman-Venkert R, White R, Lee J-K, Werman B, Krelin Y (2004). The precursor form of IL-1α is an intracrine proinflammatory activator of transcription. Proc Natl Acad Sci U S A.

[23] Gilbert F, Cunha P, Jensen K, Glass E, Foucras G, Robert-Granie C (2013). Differential response of bovine mammary epithelial cells to Staphylococcus aureus or Escherichia coli agonists of the innate immune system. Vet Res.

[24] Chen T, Li E (2006). Establishment and maintenance of DNA methylation patterns in mammals. Curr Top Microbiol Immunol.

[25] Arita K, Ariyoshi M, Tochio H, Nakamura Y, Shirakawa M (2008). Recognition of hemi-methylated DNA by the SRA protein UHRF1 by a base-flipping mechanism. Nature.

[26] Hashimoto H, Horton JR, Zhang X, Bostick M, Jacobsen SE, Cheng X (2008). The SRA domain of UHRF1 flips 5-methylcytosine out of the DNA helix. Nature.

[27] O’Gorman A, Colleran A, Ryan A, Mann J, Egan LJ (2010). Regulation of NF-κB responses by epigenetic suppression of IκBα expression in HCT116 intestinal epithelial cells. Am J Physiol Gastrointest Liver Physiol.

[28] Bock C, Walter J, Paulsen M, Lengauer T (2008). Inter-individual variation of DNA methylation and its implications for large-scale epigenome mapping. Nucleic Acids Res.

[29] Shen H, Qiu C, Li J, Tian Q, Deng H-W (2013). Characterization of the DNA methylome and its interindividual variation in human peripheral blood monocytes. Epigenomics.

[30] Harris RA, Wang T, Coarfa C, Nagarajan RP, Hong C, Downey SL (2010). Comparison of sequencing-based methods to profile DNA methylation and identification of monoallelic epigenetic modifications. Nat Biotech.

[31] Sun L, Gong Z, Oberst EJ, Betancourt A, Adams AA, Horohov DW (2013). The promoter region of interferon-gamma is hypermethylated in neonatal foals and its demethylation is associated with increased gene expression. Developmental & Comparative Immunology.

[32] Stefani FA, Viana MB, Dupim AC, Brito JAR, Gomez RS, da Costa JE (2013). Expression, polymorphism and methylation pattern of interleukin-6 in periodontal tissues. Immunobiology.

[33] Raddatz G, Hagemann S, Aran D, Sohle J, Kulkarni P, Kaderali L (2013). Aging is associated with highly defined epigenetic changes in the human epidermis. Epigenetics Chromatin.

[34] Martino D, Prescott S (2011). Epigenetics and prenatal influences on asthma and allergic airways disease. Chest.

[35] Jones PA (2012). Functions of DNA methylation: islands, start sites, gene bodies and beyond. Nature Review Genetics.

[36] Tsai H-C, Li H, Van Neste L, Cai Y, Robert C, Rassool Feyruz V (2012). Transient low doses of DNA-demethylating agents exert durable antitumor effects on hematological and epithelial tumor cells. Cancer Cell.

[37] Duijkers FM, Menezes R, Goossens-Beumer I, Stumpel DPM, Admiraal P, Pieters R (2013). Epigenetic drug combination induces genome-wide demethylation and altered gene expression in neuro-ectodermal tumor-derived cell lines. Cell Oncol.

[38] Benjamini Y, Hochberg Y (1995). Controlling the false discovery rate: a practical and powerful approach to multiple testing. J Roy Stat Soc Ser B Methodol.

[39] Krzywinski M, Schein J, Birol İ, Connors J, Gascoyne R, Horsman D (2009). Circos: an information aesthetic for comparative genomics. Genome Res.

[40] Sohn EJ, Paape MJ, Bannerman DD, Connor EE, Fetterer RH, Peters RR (2007). Shedding of sCD14 by bovine neutrophils following activation with bacterial lipopolysaccharide results in down-regulation of IL-8. Vet Res.

[41] Pareek R, Wellnitz O, Van Dorp R, Burton J, Kerr DE (2005). Immunorelevant gene expression in LPS-challenged bovine mammary epithelial cells. J Appl Genet.

[42] Bougarn S, Cunha P, Gilbert FB, Harmache A, Foucras G, Rainard P (2011). Staphylococcal-associated molecular patterns enhance expression of immune defense genes induced by IL-17 in mammary epithelial cells. Cytokine.

