# Supplementary material for: Age dependent changes in the LPS induced transcriptome of bovine dermal fibroblasts occurs without major changes in the methylome
Source: BMC Genomics. 2015 Jan 27;16(1):30. doi: 10.1186/s12864-015-1223-z (PMC4312471; doi:10.1186/s12864-015-1223-z)
Supplement: Additional file 1: — Genes displaying differential expression (FDR < 0.05; CPM > 1; 2 < FC < −2) due to LPS at hour 2 as compared to hour 0. A positive fold change indicates higher expression at hour 2 than at hour 0. CPM = Counts per Million. FDR = False discovery rate. Data shown for comparisons with FDR < 0.05; CPM > 1, and fold change > 2. [file 12864_2015_1223_MOESM1_ESM.pdf]

Supplemental File 1. Genes displaying differential expression due to LPS at hour 2 as compared to hour 0. A positive fold change indicates higher expression at hour 2 than at hour 0. CPM = Count per million. FDR = False Discovery Rate.

Gene	Chromosome	Fold Change	CPM	FDR
CX3CL1	18	3415.9	1.94	2.96E-04
CCL20	2	2034	1.16	7.84E-07
CD83	23	1290.9	3.84	6.90E-04
RRAD	18	518	1.42	6.59E-04
FOSB	18	203.8	25.13	3.84E-05
ARC	14	200.2	2.59	2.79E-04
TNF	23	125.2	2.25	1.00E-05
IL8	6	123.7	18.19	1.81E-05
EGR3	8	105.5	5	1.72E-04
C2CD4B	10	99.1	1.44	8.44E-05
RND1	5	91.9	38.9	8.73E-04
IL1A	11	86	1.51	5.90E-04
IL6	4	83	11.32	9.42E-04
XIRP1	22	59.8	5.66	4.57E-04
LIF	17	55.9	18.71	6.39E-05
TNFAIP3	9	44.4	17.44	6.93E-04
C28H10orf10	28	44.2	1.1	7.29E-04
CXCL2	6	43.8	97.37	6.91E-04
NR4A1	5	41	79.93	4.84E-04
SLCO4A1	13	36.7	1.3	4.00E-04
NFKBIA	21	36.1	98.66	4.50E-04
CCL5	19	35.4	30.74	3.55E-05
PTX3	1	35.3	2.91	7.31E-04
ID1	13	32.2	65.19	5.92E-04
RGS16	16	30.9	2.44	1.97E-04
CCL2	19	28.5	164.54	4.33E-04
DUSP5	26	27.8	5.3	9.30E-04
F3	3	25.1	55.12	3.58E-04
NFKBIZ	1	23.8	58.98	5.89E-04
PTGS2	16	20.5	65.35	6.77E-04
LOC100138376	16	20.3	39.4	3.44E-04
MAP3K8	13	19.2	6.55	8.21E-04
EGR2	28	17.3	1.05	2.40E-04
SAMD11	16	14.6	9.76	2.39E-05
DLX3	19	14.4	1.58	1.32E-04
HAS2	14	13.2	3.87	6.78E-04
LOC100848038	7	12.8	131.33	6.93E-04
ZC3H12A	3	12.6	25.93	5.69E-04

IER3	23	12.4	268.36	7.46E-04
FOSL1	29	11.8	52.22	5.28E-04
IRF1	7	11.5	53.42	2.32E-04
CFB	23	10.8	7.21	7.21E-04
SMAD7	24	10.3	23.38	9.20E-04
NFATC1	24	10	73.94	2.40E-04
N4BP3	7	9.8	6.75	1.34E-04
TMEM88	19	9.5	1.51	8.71E-04
CSRNP1	22	9	24.23	1.49E-04
PIM1	23	8.4	47.45	5.36E-04
JUNB	7	8	99.16	8.55E-04
CDA	2	7.9	1.39	5.23E-04
HEY2	9	7.8	2.24	5.99E-04
HEY1	14	7.5	2.84	3.29E-04
MAFF	5	7.4	14.26	8.81E-04
CCDC85B	29	7.2	26.46	4.00E-05
DUSP6	5	7	15.01	1.63E-04
MARCKSL1	2	6.8	10.41	6.31E-04
SCXA	14	6.7	1.12	3.77E-04
SNAI1	13	6.7	13.37	5.09E-04
LFNG	25	6.7	63.5	6.58E-04
HBEGF	7	6.6	3.74	3.05E-04
CXCR4	2	6.6	1.16	7.08E-04
LOC782264	12	6.4	2.78	1.55E-04
EDN1	23	6.3	1.28	5.89E-04
BIRC3	15	6.1	39.43	1.44E-04
LOC100847884	4	5.9	3.03	7.48E-04
NUAK2	16	5.5	22.09	8.92E-05
ATP8A2	12	5.5	6.19	7.46E-04
LOC100337435	21	5.4	26.13	1.62E-04
ARRDC2	7	5.4	26.09	2.43E-04
RNF19B	2	5.1	25.72	4.79E-04
KLF10	14	5	102.84	9.74E-04
LOC100847310	18	4.9	68.53	7.22E-04
PLAUR	18	4.9	63.33	8.03E-04
GJC1	19	4.7	3.5	8.53E-04
ERRFI1	16	4.6	76.47	8.70E-04
VASN	25	4.5	106.99	6.79E-04
PLK3	3	4.4	28.23	2.25E-04
IER2	7	4.4	36.13	4.65E-04
SKIL	1	4.3	31.48	6.12E-05
WNT10B	5	4.3	4.21	8.45E-05
NFKBID	18	4.3	6.15	3.90E-04
C23H6orf145	23	4.3	43.24	6.94E-04
GADD45B	7	4.3	81.95	9.41E-04
NAB2	5	4.2	62.47	5.75E-04

KLHL21	16	4.2	151.42	9.11E-04
PHF21B	5	4.1	2.16	6.95E-04
ELFN1	25	4.1	1	0.001
ZSWIM4	7	4	10.2	6.45E-05
NEFH	17	4	3.62	5.03E-04
SDC4	13	4	109.66	6.18E-04
METRNL	19	4	72.82	7.58E-04
HIVEP3	3	3.9	2.12	7.23E-05
RELB	18	3.9	18.07	2.89E-04
BCL2L11	11	3.9	1.98	5.41E-04
PDE4B	3	3.9	5.12	6.88E-04
C10H14orf43	10	3.9	26.13	9.57E-04
C15H11orf96	15	3.9	73.62	9.78E-04
CLCF1	29	3.8	3.36	1.23E-04
GPC6	12	3.8	22.79	3.93E-04
PHLDA1	5	3.8	8.6	6.48E-04
DLX2	2	3.8	4.97	7.32E-04
LOC100336535	19	3.8	2.3	8.91E-04
KDM6B	19	3.7	39.04	2.64E-04
CORO7	25	3.7	98.49	4.34E-04
CXXC5	7	3.7	48.81	4.62E-04
SPSB1	16	3.7	21.25	4.96E-04
RNF125	24	3.7	4.25	5.78E-04
LOC100849059	7	3.7	2.02	6.40E-04
MT2A	18	3.7	14.89	8.26E-04
SERTAD1	18	3.6	44.93	5.83E-04
DNAH2	19	3.6	1.21	7.22E-04
SERPINB2	24	3.6	1.12	8.32E-04
CREB5	4	3.5	1.62	2.82E-05
HCN4	10	3.5	1.62	4.73E-05
TBX3	17	3.5	6.93	1.32E-04
ZFAND2A	25	3.4	3.52	1.65E-04
TNIP1	7	3.4	100.27	1.72E-04
BCL3	18	3.4	40.83	3.97E-04
TBX2	19	3.4	1.57	6.41E-04
BTG2	16	3.4	23.12	9.95E-04
ERF	18	3.4	21.07	9.95E-04
HES4	16	3.4	1.2	0.001
CD40	13	3.3	8.26	1.31E-04
PIM3	5	3.3	10.28	8.12E-04
CCRN4L	17	3.2	19.48	4.03E-04
AXIN2	19	3.2	10.85	5.32E-04
NR4A3	8	3.2	10.3	0.006
MIDN	7	3.1	27.97	4.65E-04
JHDM1D	4	3.1	3.22	7.71E-04

EPHA2	2	3.1	35.41	9.60E-04
SYNJ2	9	3	6.56	7.69E-04
HMGA1	23	3	19.13	7.92E-04
CISH	22	3	1.55	8.97E-04
NFKB1	6	2.9	23.23	9.27E-05
TRIB1	14	2.9	12.33	1.35E-04
ARL4C	3	2.9	21.93	1.51E-04
IER5L	11	2.9	21.41	1.52E-04
PAPD7	20	2.9	33.23	2.67E-04
TICAM1	7	2.9	14.04	5.27E-04
TNFSF9	7	2.9	30.1	6.59E-04
SMAD6	10	2.9	19.66	6.79E-04
ARID5A	11	2.9	7.8	8.72E-04
BHLHE40	22	2.8	248.99	9.10E-05
C12H13orf15	12	2.8	57.61	2.62E-04
HEYL	3	2.8	3.86	9.61E-04
NFKBIE	23	2.8	13.07	9.82E-04
OLR1	5	2.8	12.31	0.015
GRM8	4	2.7	2.4	6.44E-05
LOC785529	7	2.7	4.15	2.10E-04
BCOR	X	2.7	34.16	4.69E-04
ETS1	29	2.7	47.2	8.68E-04
LOC100298963	17	2.7	1.23	0.002
KLK10	18	2.7	2.43	0.006
BCAR1	18	2.6	63.54	2.94E-04
ITPRIP	26	2.6	22.33	3.05E-04
CD3EAP	18	2.6	12.02	3.85E-04
TNFAIP8L3	10	2.6	6.2	5.92E-04
TMEM158	22	2.6	10.84	8.37E-04
ID2	11	2.6	83.1	8.44E-04
TRIM47	19	2.6	11.36	9.27E-04
OSGIN1	18	2.6	2.91	9.89E-04
CRIP1	21	2.6	259.57	0.02
TBC1D10A	17	2.5	58.03	1.85E-04
GATA2	22	2.5	20.74	2.41E-04
TACR1	11	2.5	1.33	3.72E-04
LOC100848679	4	2.5	5.61	6.53E-04
PFKFB3	13	2.5	12.18	9.65E-04
LOC510442	7	2.4	33.6	4.18E-05
RIN2	13	2.4	12.61	7.88E-05
RASGEF1A	28	2.4	1.06	1.56E-04
SLC2A3	5	2.4	55.6	1.73E-04
PMEPA1	13	2.4	16.44	2.76E-04
JUND	7	2.4	56.58	4.15E-04
SERPINE1	25	2.4	2855.75	4.18E-04

NAB1	2	2.4	24.69	4.85E-04
TOB2	5	2.4	43.8	5.83E-04
ULBP1	9	2.4	12.66	6.82E-04
MSX1	6	2.4	6.08	8.63E-04
HMOX1	5	2.4	54.99	9.16E-04
MUC12	25	2.3	2649.24	1.05E-04
CSPG4	21	2.3	95.06	5.01E-04
SHROOM3	6	2.3	9.78	6.57E-04
PRKX	X	2.3	9.48	6.84E-04
PDLIM4	7	2.3	44.69	9.60E-04
CXCL6	6	2.3	68.38	0.001
GPR132	21	2.3	3.91	0.001
PTGES	11	2.3	11.16	0.001
SLC35E4	17	2.3	4.77	0.001
IRF7	29	2.3	9.35	0.002
ADPRHL1	12	2.3	2.72	0.01
TNFAIP6	2	2.3	10.38	0.013
EGR1	7	2.3	9.65	0.018
LOC100335751	6	2.3	3.37	0.025
JUN	3	2.2	36.91	1.09E-05
FSTL3	7	2.2	60.17	1.45E-05
MAP3K11	29	2.2	12.22	9.75E-05
GRASP	5	2.2	6.44	3.26E-04
MFSD2A	3	2.2	25.51	6.07E-04
C21H15orf39	21	2.2	16.46	6.99E-04
PPP1R13L	18	2.2	18.33	7.56E-04
ZNF469	18	2.2	27.65	7.81E-04
FAM89B	29	2.2	14.19	8.15E-04
LOC100848808	4	2.2	4.8	8.87E-04
IER5	16	2.2	27.67	8.95E-04
SLC20A1	11	2.2	80.36	0.001
LY6G5B	23	2.2	1.03	0.023
LOC100847452	4	2.2	2.95	0.025
NEDD9	23	2.2	19.96	0.039
MAP2K3	19	2.1	71.01	4.85E-05
ATF3	16	2.1	15.87	3.24E-04
ZNF574	18	2.1	14.98	4.98E-04
TSKU	15	2.1	54.54	5.89E-04
PCSK7	15	2.1	87.39	9.43E-04
TSHZ3	18	2.1	12.26	9.56E-04
LOC100337268	18	2.1	5.4	9.81E-04
LOC100849045	24	2.1	14.92	0.001
LRRC32	15	2.1	22.21	0.001
MAFK	25	2.1	6.04	0.001
NOG	19	2.1	3.7	0.001

SGMS2	6	2.1	2.76	0.001
SIK1	1	2.1	19.43	0.001
ETV3	3	2.1	9.78	0.004
GCH1	10	2.1	5.1	0.006
GJB3	3	2.1	1.07	0.017
CPEB2	6	2.1	25.54	0.038
LOC100294787	22	2.1	1.51	0.044
SNAI2	14	2	187.01	1.92E-04
TCF7	7	2	7.35	2.69E-04
INSIG1	4	2	69.86	4.25E-04
FLT1	12	2	27.03	6.33E-04
HSPA1A	23	2	87.54	8.67E-04
FAM20A	19	2	11.47	0.001
FHDC1	17	2	7.64	0.001
VPS37B	17	2	9.44	0.001
IRF5	4	2	3.35	0.003
PLEKHO1	3	2	35.18	0.008
CDC42EP2	29	2	4.45	0.026
RMI1	8	-2	5.54	1.91E-04
LOC100847448	20	-2	65.36	9.67E-04
LOC617476	18	-2	2.81	0.001
ZNF566	18	-2	2.16	0.002
ZNF862	4	-2	1.46	0.002
ZNF48	25	-2	1.89	0.003
FAM84B	14	-2	14.19	0.007
ZNF512	11	-2	14.42	0.008
FBXL8	18	-2	1.51	0.011
ITGA10	3	-2	1.03	0.013
CCNG2	6	-2	5.93	0.015
PLA2G4B	10	-2	1.04	0.026
LOC100848248	11	-2	1.56	0.037
SMARCAD1	6	-2	15.98	0.042
ZSCAN21	25	-2.1	7.7	7.50E-05
C4H7orf25	4	-2.1	6.73	9.00E-05
ZNF792	18	-2.1	3.6	1.39E-04
ZNF174	25	-2.1	4.35	4.54E-04
DPH2	3	-2.1	4.75	6.14E-04
FBXL12	7	-2.1	6.22	6.23E-04
LOC100847474	23	-2.1	22.77	9.27E-04
ZSCAN16	23	-2.1	1.57	9.82E-04
LOC618337	18	-2.1	2.51	0.001
PPP1R10	23	-2.1	20.78	0.001
ZNF286A	19	-2.1	2.59	0.001
ZNF75A	25	-2.1	3.06	0.001
GLI1	5	-2.1	1.82	0.002

RIN1	29	-2.1	3.12	0.002
ZBTB38	1	-2.1	13.32	0.002
LOC100847683	15	-2.1	1.41	0.003
ZBTB7C	24	-2.1	1.58	0.003
ZNF585A	18	-2.1	1.32	0.003
NUAK1	5	-2.1	56.4	0.005
ARMCX4	X	-2.1	1.94	0.006
XPNPEP3	5	-2.1	1.38	0.012
CBFA2T2	13	-2.1	4.01	0.016
JRKL	15	-2.1	2.83	0.017
LIPT1	11	-2.2	1.91	7.00E-05
RAP2B	1	-2.2	19.94	2.27E-04
ZNF181	18	-2.2	3.21	5.05E-04
ZNF540	18	-2.2	13.63	5.48E-04
LOC100301148	18	-2.2	4.09	7.44E-04
RASL11B	6	-2.2	2.92	7.45E-04
NR1D1	19	-2.2	7.49	8.04E-04
MARS2	2	-2.2	1.66	9.00E-04
ZNF135	18	-2.2	1.46	9.43E-04
ZNF691	3	-2.2	3.34	9.87E-04
ZNF140	17	-2.2	1.96	0.001
ZNF182	X	-2.2	1.37	0.001
ZNF200	25	-2.2	4.49	0.001
ZNF26	17	-2.2	2.64	0.001
ZSCAN12	23	-2.2	1.9	0.001
MBLAC2	7	-2.2	2.3	0.002
NUDT18	8	-2.2	2.76	0.002
RAPGEFL1	19	-2.2	1.29	0.006
ORC2	2	-2.2	10.21	0.014
ZNF280C	X	-2.2	1.63	0.018
TRIM23	20	-2.2	3.63	0.043
CH25H	26	-2.3	8.3	1.18E-04
OSR1	11	-2.3	4.81	4.55E-04
TRIB3	13	-2.3	31.77	4.82E-04
ZNF354C	7	-2.3	1.56	6.40E-04
ZFP30	18	-2.3	6.84	8.46E-04
LOC785831	18	-2.3	1.46	0.001
FAM110A	13	-2.3	1.42	0.002
SENP8	10	-2.3	1.67	0.003
SOX13	16	-2.3	1.1	0.003
ZNF167	22	-2.3	1.09	0.003
KIF18A	15	-2.3	2.37	0.01
ZNF646	25	-2.4	5.03	6.19E-05
ZNF93	18	-2.4	2.76	4.82E-04
OXSM	27	-2.4	2.58	4.93E-04

ZNF260	18	-2.4	3.73	6.15E-04
TNFSF18	16	-2.4	62.67	7.66E-04
SGOL1	1	-2.4	2.61	0.001
ZBTB40	2	-2.4	2.57	0.008
LOC528802	18	-2.4	3.02	0.022
AMIGO1	3	-2.5	1.53	3.76E-04
KIAA1383	28	-2.5	1.03	8.03E-04
ZNF850	18	-2.5	7.34	9.95E-04
ZNF211	18	-2.5	1.91	0.005
PIK3R1	20	-2.5	38.51	0.006
LOC616903	8	-2.5	2.84	0.015
WDR5B	1	-2.6	2.24	2.53E-04
ZNF624	19	-2.6	2.66	3.52E-04
LOC790312	10	-2.6	19.59	3.85E-04
C16H1orf74	16	-2.6	3	4.02E-04
ZNF22	28	-2.6	3.35	4.23E-04
IRX3	18	-2.6	7.7	5.43E-04
PSRC1	3	-2.6	2.35	6.68E-04
ZNF43	18	-2.6	2.78	9.63E-04
LCMT2	21	-2.7	1.45	6.45E-05
ZFHX2	10	-2.7	1.15	1.21E-04
ZNF572	14	-2.7	5.56	2.50E-04
SUV420H2	18	-2.7	1.4	4.46E-04
MYLK3	18	-2.7	1.13	7.67E-04
C11H2orf42	11	-2.7	3.54	8.62E-04
CASP8AP2	9	-2.7	6.78	0.004
FLRT3	13	-2.8	7.08	1.80E-04
ZNF323	23	-2.8	1.55	2.84E-04
GAS1	8	-2.8	3.5	4.51E-04
ZFP62	7	-2.8	1.08	4.63E-04
ZNF398	4	-2.8	1.89	5.70E-04
LOC100848182	18	-2.8	2.88	8.05E-04
SETMAR	22	-2.8	2.87	9.20E-04
PAX6	15	-2.8	2.52	0.001
KIF18B	19	-2.9	3.11	9.21E-04
ZKSCAN2	25	-2.9	1.03	0.004
ZNF689	25	-2.9	2.1	0.004
PLK2	20	-3	77.2	1.52E-04
ZNF248	28	-3	2.38	3.33E-04
ZNF202	15	-3	1.45	6.25E-04
ZNF548	18	-3	1.75	9.53E-04
RND3	2	-3.1	163.76	8.83E-05
EHHADH	1	-3.1	1.3	6.57E-04
C11H2orf44	11	-3.1	2.41	9.31E-04
ZNF770	10	-3.2	2.66	4.58E-05

ZSCAN20	3	-3.2	1.52	1.37E-04
SIX1	10	-3.2	4.83	4.83E-04
LOC100299025	18	-3.2	1.18	7.64E-04
ZNF596	1	-3.2	3.44	8.42E-04
CDKN2B	8	-3.2	68.24	9.81E-04
FAM46A	9	-3.2	9.21	0.024
ZNF570	18	-3.5	2.14	2.79E-04
ZNF397	24	-3.5	1.79	4.60E-04
CCDC142	11	-3.6	1.26	5.78E-04
ARRDC4	21	-3.6	5.37	0.005
LOC100849024	22	-3.7	1.57	1.62E-04
JUB	10	-3.7	5.9	7.21E-04
ZNF2	11	-3.7	2.01	8.51E-04
C3H1orf51	3	-3.8	1.42	1.96E-04
IRX5	18	-3.9	8.72	3.66E-04
SGK1	9	-4.3	44.35	7.74E-05
ARRDC3	7	-4.4	8.86	9.65E-04
LRIG3	5	-4.6	26.54	4.43E-04
ZNF319	18	-4.7	1.03	6.29E-04
CHAC1	10	-5.6	12.63	9.03E-05
SPRY1	17	-6	5.3	3.32E-04
KCNJ2	19	-12.2	2.39	6.73E-04
KCNE4	2	-15.3	12.85	8.37E-04
TXNIP	3	-16.6	29.34	2.46E-04
LOC100848155	3	-18	3.63	1.23E-04
