# Supplementary material for: Age dependent changes in the LPS induced transcriptome of bovine dermal fibroblasts occurs without major changes in the methylome
Source: BMC Genomics. 2015 Jan 27;16(1):30. doi: 10.1186/s12864-015-1223-z (PMC4312471; doi:10.1186/s12864-015-1223-z)
Supplement: Additional file 2: — Genes displaying differential expression (FDR < 0.05; CPM > 1; 2 < FC < −2) due to LPS at hour 8 as compared to hour 0. A positive fold change indicates higher expression at hour 8 than at hour 0. CPM = Counts per Million. FDR = False discovery rate. Data shown for comparisons with FDR < 0.05; CPM > 1, and fold change > 2. [file 12864_2015_1223_MOESM2_ESM.pdf]

Supplemental File 2. Genes displaying differential expression due to LPS at hour 8 as compared to hour 0. A positive fold change indicates higher expression at hour 8 than at hour 0. CPM = Counts per Million. FDR = False discovery rate.

Gene	Chromosome	Fold Change	CPM	FDR
SAA3	29	2498.3	1.44	1.41E-06
IL8	6	273.7	32.27	7.40E-06
IL6	4	131.6	18.84	4.97E-06
MX2	1	121.2	1.04	4.85E-06
CFB	23	87.7	42.26	7.89E-05
ISG15	16	63.2	2.61	1.09E-05
CCL5	19	48.4	22.41	1.15E-05
PTX3	1	43.1	2.56	1.52E-05
PDPN	16	41.6	1.09	2.26E-05
CXCL2	6	27.1	52.25	2.48E-05
CCL2	19	26.7	121	3.84E-05
OAS2	17	26.1	2.4	9.07E-05
OAS1	17	24.6	17.46	4.05E-05
SERPINB2	24	23	5.99	4.18E-05
TGM3	13	22.2	6.88	4.36E-05
RND1	5	20.2	6.04	5.60E-05
ZBP1	13	18.8	1.05	5.62E-05
CLDN11	1	17.7	1.42	6.30E-05
TNFAIP3	9	15.9	5.53	6.82E-05
IFIH1	2	15.1	3.02	7.15E-05
HAS2	14	12.7	3.83	7.33E-05
CXCL6	6	12.2	301.64	7.66E-05
NFKBIA	21	10	29.41	1.08E-05
OCLN	20	9.1	2.11	8.04E-05
SAMD11	16	8.7	6.34	3.84E-05
C2	23	8.5	1.23	9.21E-05
STRA6	21	8.4	1.03	9.31E-05
ID1	13	7.8	17.9	9.39E-05
CDA	2	7.7	1.37	9.69E-05
TLR2	17	6.8	1.52	9.72E-05
COL11A2	23	6.6	1.1	9.90E-05
LOC100138376	16	6.6	11.05	0.000100505
PDE4B	3	6.6	8.52	0.000100604
PTGS2	16	6.5	17.93	0.000102617
CYP7B1	14	5.9	3.12	0.000102655
CMPK2	11	5.6	1.54	0.000102848
CD40	13	5.4	12.23	0.000113075

OLR1	5	5.4	17.86	0.000114377
F3	3	5.2	13.14	0.000115567
PION	4	5.1	1.21	0.000127029
LOC540363	17	5	1.04	0.000130481
MYO5C	10	4.8	1.08	0.000130524
PTGS1	11	4.8	31.37	0.00013146
CYP3A4	25	4.6	26.76	0.000140791
SV2C	10	4.5	2.06	0.000154462
IL36A	11	4.4	3.68	0.000154794
BST2	7	4.3	14.97	0.000155203
IFI27	21	4.3	2.31	0.000169123
LIF	17	4.3	1.78	0.000172245
LOC100297676	5	4.3	15.9	0.000172826
MX1	1	4.3	16.18	0.000174458
BIRC3	15	4.1	27.12	0.000194924
NFKBIZ	1	4.1	10.21	0.000196753
LOC100336368	4	3.8	1.78	0.000204982
MT2A	18	3.8	8.84	0.000210461
NEFH	17	3.7	3.91	0.000211344
SMAD9	12	3.7	4.9	0.000218784
ADAMTS6	20	3.6	7.4	0.000219001
LOC100848019	9	3.6	33.27	0.000221091
GFPT2	7	3.5	127.52	0.000224119
MXRA5	X	3.5	9.73	0.000229365
PLA2G4A	16	3.5	38.86	0.000231733
TNIP1	7	3.5	93.28	0.000243884
FILIP1	9	3.3	2.34	0.000245985
LOC100847310	18	3.3	56.3	0.000249448
PLAUR	18	3.3	52.02	0.000252933
F2R	10	3.2	2.27	0.000259744
GALNTL2	1	3.2	2.34	0.000268808
IRF1	7	3.2	17.29	0.000271687
LOC100848038	7	3.2	38.15	0.000284955
ARNTL	15	3.1	21.69	0.00029058
CPM	5	3.1	7.53	0.000292164
HEY1	14	3.1	1.6	0.000293673
LOC782264	12	3.1	1.69	0.000299088
MAP3K8	13	3.1	1.19	0.00030475
MARCKSL1	2	3.1	5.69	0.0003087
NCALD	14	3.1	1.31	0.000309616
TNFAIP8L3	10	3.1	7.37	0.000310696
ALDH1A3	21	3	56.79	0.0003191
ANGPTL4	7	3	15.83	0.000320317
CDC6	19	3	3.17	0.00032721
RTP4	1	3	2.79	0.000327895

CA2	14	2.9	60.95	0.000339456
SH3GLB1	3	2.9	43.65	0.000339496
IL1RL1	11	2.8	28.33	0.000340183
MTSS1	14	2.8	1.14	0.000345442
ATP8A2	12	2.7	3.62	0.000351864
FOSL1	29	2.7	17.19	0.000363231
FXYD6	15	2.7	1.63	0.000364387
GPC6	12	2.7	17.36	0.000366825
HEY2	9	2.7	1.02	0.000369222
MAFF	5	2.7	6.66	0.00037337
PDE3A	5	2.7	14.52	0.000376005
PMAIP1	24	2.7	31.5	0.000376543
SDPR	2	2.7	2.31	0.000376799
SIPA1L2	28	2.7	12.66	0.000377815
SOD2	9	2.7	8.6	0.0003859
ARSI	7	2.6	4.38	0.000393792
BDKRB2	21	2.6	3.32	0.000400431
GLDC	8	2.6	12.24	0.000401791
EPSTI1	12	2.5	8.05	0.000403938
PCDH11Y	X	2.5	39.72	0.000410484
PDXK	1	2.5	18.45	0.000412392
PHLDA1	5	2.5	6.58	0.00041776
PTGFR	3	2.5	7.8	0.000421236
RELB	18	2.5	12.56	0.000421896
RNF125	24	2.5	2.96	0.000424891
SMAD7	24	2.5	7.31	0.000437219
DDC	4	2.4	1.37	0.000446223
DHX58	19	2.4	3.67	0.000448662
ESM1	20	2.4	48.54	0.000453301
IL15RA	13	2.4	14	0.000456898
PNLDC1	9	2.4	1.21	0.000457979
VAMP1	5	2.4	4.06	0.000473182
ADAMTS3	6	2.3	3.56	0.000477368
AMACR	20	2.3	18.23	0.00048683
BDKRB1	21	2.3	5.47	0.000496177
COL27A1	8	2.3	4.83	0.000501848
EFNB2	12	2.3	1.77	0.000502053
FAM20A	19	2.3	12.86	0.000502973
FLT1	12	2.3	31.73	0.000503774
NEDD4L	24	2.3	86.79	0.000504932
RAB20	12	2.3	4.04	0.000509464
SKIL	1	2.3	18.77	0.000513327
BDNF	15	2.2	2.4	0.000513524
EDNRA	17	2.2	12.02	0.000513847
KLK10	18	2.2	2.23	0.000518864

LOC508347	3	2.2	2.22	0.000520384
PHF21B	5	2.2	1.47	0.000520622
PTPRU	2	2.2	6.71	0.000528989
RGS17	9	2.2	14.52	0.000532981
SRM	16	2.2	37.33	0.00053466
TGFBI	7	2.2	10.26	0.000542327
TMEM158	22	2.2	9.89	0.000544761
ZC3H12A	3	2.2	6.28	0.000544769
CCBE1	24	2.1	6.12	0.000545894
CDCA7	2	2.1	6.38	0.00054755
ENOX1	12	2.1	11.67	0.00055562
ERRFI1	16	2.1	45.64	0.000559578
ITGA2	20	2.1	2.9	0.000561785
KCNJ5	29	2.1	8.92	0.000562694
MB21D1	9	2.1	2.59	0.000568659
NFKB2	26	2.1	51.8	0.000577856
OSBPL3	4	2.1	2.68	0.00058049
SLC39A14	8	2.1	53.51	0.000582042
SOCS2	5	2.1	3.82	0.000586526
SP100	2	2.1	1.49	0.000588842
SPATA13	12	2.1	1.37	0.000590658
SYNJ2	9	2.1	5.37	0.000594082
TAP1	23	2.1	3.53	0.000597917
TSPAN12	4	2.1	3.21	0.000603764
UGDH	6	2.1	91.02	0.000607352
AGRN	16	2	1.05	0.000615694
CLMP	15	2	52.23	0.000616312
DLX2	2	2	3.3	0.000619466
IER3	23	2	43.63	0.000620621
LOC539953	16	2	9.79	0.000622093
MCTP2	21	2	4.26	0.00062627
PLXNA2	16	2	5.83	0.000634452
PROM1	6	2	7.13	0.000635678
SERPINE2	2	2	179.98	0.000637487
SLC24A2	8	2	5.23	0.000640036
SPEF2	20	2	1.1	0.000643268
ULBP1	9	2	11.18	0.000645468
ZSWIM4	7	2	6.69	0.00064646
ADAM33	13	-2	126.73	0.000647535
C13H20orf112	13	-2	1.51	0.000653969
C3H1orf51	3	-2	1.81	0.000655412
CARD10	5	-2	2.39	0.000658565
CDCA3	5	-2	8.67	0.000662776
GPR132	21	-2	1.64	0.000664251
LOC100848674	11	-2	5.24	0.000670291

LOC788842	25	-2	4.02	0.000671138
NRP2	2	-2	115.77	0.000671581
AHNAK2	21	-2.1	28.69	0.000678713
B3GALT2	16	-2.1	2.72	0.000686145
C1QTNF7	6	-2.1	1.47	0.00068689
DDX17	5	-2.1	126.84	0.000693482
EPOR	7	-2.1	2.15	0.000693583
FAM13C	28	-2.1	20.78	0.000703867
FAM64A	19	-2.1	11.7	0.000714356
FNDC4	11	-2.1	5.75	0.000716018
KLHL13	X	-2.1	3.81	0.000718901
LIMS2	2	-2.1	12.47	0.000722676
LOC100138767	18	-2.1	1.62	0.000723136
MAF	18	-2.1	8.87	0.000725698
MARCKS	9	-2.1	13.43	0.000729544
PLEKHF1	18	-2.1	9.98	0.000729734
RSPO2	14	-2.1	1.75	0.000731635
SHF	10	-2.1	13.86	0.000734296
SLC7A8	10	-2.1	1.41	0.00074289
SQLE	14	-2.1	101.14	0.000750649
TPPP	20	-2.1	4	0.000751706
TRAF5	16	-2.1	7.05	0.000757184
WDR35	11	-2.1	61.68	0.00076593
C11H9orf167	11	-2.2	1.04	0.000766257
C1H21orf62	1	-2.2	2.87	0.000767648
CA13	14	-2.2	3.2	0.000769959
CNGA3	11	-2.2	18.85	0.000788783
DMPK	18	-2.2	13.61	0.000790028
FAM46B	2	-2.2	10.97	0.000790892
HJURP	3	-2.2	12.54	0.000791121
HNMT	2	-2.2	40.91	0.000794431
ODZ3	27	-2.2	2.24	0.000794903
PPL	25	-2.2	12.85	0.00079511
QPRT	25	-2.2	4.56	0.000796174
RCOR2	29	-2.2	2.97	0.000801465
SLC6A9	13	-2.2	14.24	0.000804199
SORBS2	27	-2.2	1.61	0.000810421
TMEM173	7	-2.2	2.79	0.000826986
TYRO3	10	-2.2	33.95	0.00083664
AFAP1L2	26	-2.3	9.83	0.000837668
CCDC88B	29	-2.3	1.64	0.0008377
PAK1	29	-2.3	38.93	0.000838771
PPM1K	6	-2.3	34.02	0.000840952
C18H16orf74	18	-2.4	1.34	0.000842227
CCNG2	6	-2.4	5.89	0.000842907

CLIC5	23	-2.4	2.23	0.000849071
CYP1A1	21	-2.4	7.07	0.000852783
FBXO32	14	-2.4	2.31	0.000855044
GFRA4	13	-2.4	4.78	0.000857684
HPCAL4	3	-2.4	1.49	0.000857811
IFITM5	11	-2.4	1.14	0.000859951
JUP	19	-2.4	33.15	0.00086506
PCSK4	7	-2.4	1.39	0.000867595
SREBF1	19	-2.4	56.6	0.000868466
USP9Y	Y	-2.4	1.91	0.000872074
YPEL4	15	-2.4	1.28	0.000878247
AMIGO2	5	-2.5	35.55	0.000886383
AQP11	29	-2.5	1.85	0.000892438
BMPER	4	-2.5	1.42	0.000894115
FDFT1	8	-2.5	58.03	0.000895984
GRIK5	18	-2.5	1.78	0.000896111
HSPB2	15	-2.5	2.26	0.000896234
MMP11	17	-2.5	5.85	0.000898146
ZBTB7C	24	-2.5	1.53	0.000902349
CAPS	7	-2.6	1.44	0.000907413
DBP	18	-2.6	2.74	0.000907482
GLI1	5	-2.6	1.93	0.000910287
HMGCS1	20	-2.6	206.29	0.000916031
KANK1	8	-2.6	19.2	0.000917408
MXD3	7	-2.6	1.76	0.00091834
SESN3	15	-2.6	12.23	0.000920052
LOC616198	9	-2.7	1.39	0.000932089
MRAP2	9	-2.7	1.13	0.000932103
RAB6B	1	-2.7	1.07	0.000934983
ACSS2	13	-2.8	12.8	0.000936924
LRRN2	16	-2.8	2.55	0.00093891
ARVCF	17	-2.9	1.38	0.000939977
LPIN1	11	-2.9	3.3	0.000953046
MNT	19	-2.9	5.65	0.000955944
RAB3IL1	29	-2.9	5.03	0.000956394
LOC100847452	4	-3	1.2	0.00096123
ATF5	18	-3.2	73.16	0.000969506
PIK3IP1	17	-3.2	5.12	0.000969809
PAK6	10	-3.3	1.56	0.000979391
INSIG1	4	-3.9	31.67	0.000991481
LMCD1	22	-5	20.25	0.000999592
