# Supplementary material for: Age dependent changes in the LPS induced transcriptome of bovine dermal fibroblasts occurs without major changes in the methylome
Source: BMC Genomics. 2015 Jan 27;16(1):30. doi: 10.1186/s12864-015-1223-z (PMC4312471; doi:10.1186/s12864-015-1223-z)
Supplement: Additional file 3: — Differentially expressed genes (FDR < 0.05; CPM > 1; 2 < FC < −2) between young (5 months) and old (16 months) fibroblast cultures from the same individual exposed to 100 ng/ml LPS for 0, 2, or 8 hours. A positive fold change indicates higher expression in old cultures. CPM = Counts per Million. FDR = False discovery rate. [file 12864_2015_1223_MOESM3_ESM.pdf]

Supplemetal Table 3. Differentially expressed genes (FDR<0.05; CPM>1; 2<FC<-2) between young (5 months) and old (16 months) fibroblast cultures from the same individual exposed to 100 ng/ml LPS for 0, 2, or 8 hours. A positive fold change indicates higher expression in old cultures. CPM = Counts per Million. FDR = False discovery rate.

Hour 0

Gene	Chromosome	Fold Change	CPM	FDR
NPY1R	6	99.5	1.35	3.89E-61
GREB1	11	82.4	2.95	5.37E-66
ACAN	21	66.5	18.13	1.4E-17
DTX4	15	29.5	2.54	1.05E-72
LIX1	7	28.5	1.27	0.0000254
LOC100848478	4	24.8	78.28	0.00115
NTS	5	16.4	3.09	1.63E-40
COL8A1	1	13.3	9.25	6.6E-50
MAN1C1	2	13.3	2.23	2.69E-24
FGD5	22	13	5.83	3.08E-77
LOC516579	13	12.9	2.4	3.93E-48
SESN3	15	11.3	18.16	3.22E-60
SEMA5A	20	10.7	6.85	4.17E-27
ARHGDIB	5	10.3	1.28	7.88E-17
ZBTB7C	24	9.7	2.24	1.58E-37
AFAP1L2	26	8.3	13.62	1.05E-159
PTGIS	13	8.1	34.55	5.92E-81
CA13	14	8	4.38	7.94E-15
MASP1	1	7.9	11.73	4.89E-35
MN1	17	7.8	2.03	1.72E-28
RARRES1	1	7.7	54.51	9.33E-65
TOX	14	7.7	4.77	8.69E-49
BCAS1	13	7.2	3.68	3.52E-34
SDK2	19	7.1	2.13	4.92E-31
LOC100847493	20	6.7	1.29	9.12E-24
LOC100847495	23	6.7	4.06	5.73E-42
FES	21	6.6	9.65	8.49E-68
LOC512286	3	6.6	1.01	1.5E-13
KCNMB1	20	6.4	3.57	1.27E-35
LOC616198	9	6	2.14	7.78E-27
EXO1	16	5.8	1.53	3.55E-11
TNN	16	5.8	3.65	1.35E-16
CDC6	19	5.2	1.46	1.67E-08
LOC618369	5	5.2	9.63	5.08E-45
MGLL	22	5.2	14.48	4.1E-40
SMOC2	9	5.2	46.96	2.33E-15

ELN	25	4.9	409.49	3.08E-19
LOC613534	6	4.8	116.57	0.00526
PTPRU	2	4.8	4.83	2.53E-13
TFAP2A	23	4.8	3.92	7.86E-35
CACNA1C	5	4.7	2.14	1.04E-21
MARVELD2	20	4.6	1.06	6.72E-11
ITIH5	13	4.5	10.44	0.0064
E2F8	29	4.4	3.56	5.83E-28
CDT1	18	4.3	2.88	1.62E-23
ID1	13	4.3	4.71	1.77E-30
IL1RL1	11	4.2	15.12	5.5E-26
PERP	9	4.2	6.23	1.54E-22
LSP1	29	4.1	6.29	2.95E-34
CLSPN	3	4	3.61	2.8E-21
CPZ	6	4	3.46	4.07E-21
CRTAC1	26	4	1.84	2.11E-18
DAAM2	23	4	15.73	1.12E-23
EGLN3	21	4	9.03	4.02E-55
HSD17B14	18	4	2.31	1.9E-10
KITLG	5	4	2.31	3.09E-15
LOC789200	29	4	1.04	4.81E-11
PRSS48	17	4	1.1	0.00062
RRM2	11	4	48.67	4.09E-83
COL15A1	8	3.9	220.29	4.51E-59
BDKRB1	21	3.8	3.09	1.04E-08
CYP1A1	21	3.8	9.51	6.33E-54
GPR132	21	3.8	2.14	8.81E-11
PTPN3	8	3.8	4.9	5.4E-28
ITGA7	5	3.7	14.54	0.0000596
KCNMA1	28	3.7	4.73	5.46E-23
KIF1A	3	3.7	1.97	5.85E-10
MOCOS	24	3.7	2.97	1.22E-20
ADAMTS16	20	3.6	1.95	2.19E-16
LOC100336535	19	3.6	1.07	0.000000318
PLCE1	26	3.6	1.5	1.07E-12
TCF19	23	3.6	4.86	1.43E-28
CARD11	25	3.4	4.04	1.79E-10
CD34	16	3.4	4.08	2.16E-13
F13A1	23	3.4	431.33	3.59E-39
FOXM1	5	3.4	3.77	1.29E-22
ST8SIA1	5	3.4	10.88	2.89E-38
UHRF1	7	3.4	14.74	4.98E-67
CCL2	19	3.3	8.46	3.78E-23
CRYAB	15	3.3	43.97	4.21E-56
FAM49A	11	3.3	2.33	2.9E-15

IL1RL2	11	3.3	1.02	0.00128
MEOX2	4	3.3	6.19	2.12E-24
P2RY1	1	3.3	1.24	1.96E-09
CD1D	3	3.2	2.95	0.0000125
CDCA5	29	3.2	3.46	6.67E-13
HHIPL1	21	3.2	6.92	8.58E-33
MYBL1	14	3.2	4.01	1.72E-18
PTPRD	8	3.2	10.4	3.71E-30
TSTD1	3	3.2	3.27	4.75E-16
FAM105A	20	3.1	2.23	1.09E-08
FAM64A	19	3.1	15.3	1.97E-56
OLFML1	15	3.1	4.78	0.000253
ASF1B	7	3	4.64	6.7E-20
ASPM	16	3	26.59	8.19E-56
CCNF	25	3	10.72	3.47E-44
CDC20	3	3	23.93	3.86E-63
DACT2	9	3	4.1	6.9E-09
DEPDC1	3	3	5.4	1.69E-22
ELMO3	18	3	1.03	0.000000226
FAM83D	13	3	5.13	1.15E-20
GIMAP4	4	3	1.21	0.0000583
IQGAP3	3	3	21.51	5.37E-53
KIF20A	7	3	46.55	4.47E-41
LOC790886	16	3	5.35	5.94E-15
MCM5	5	3	22.23	9.06E-63
SAMD11	16	3	1.46	0.00663
VAT1L	18	3	14.14	0.0000934
CDC25B	13	2.9	16.25	1.29E-43
CFH	16	2.9	53.87	1.96E-37
CTSO	17	2.9	1.13	0.00000693
HJURP	3	2.9	16.59	1.23E-44
KIAA0101	10	2.9	10.48	2.37E-41
LOC781004	16	2.9	3.87	1.95E-15
LOC790886	16	2.9	2.17	1.78E-11
MAPK12	5	2.9	4.27	1.36E-13
MCM2	22	2.9	20.59	2.54E-53
MCM3	23	2.9	17.04	8.92E-46
MFSD6	2	2.9	7.65	1.7E-27
MT2A	18	2.9	3.49	1.8E-11
PRUNE2	8	2.9	20.14	1.37E-33
PTPRB	5	2.9	1.67	0.0000769
SLC25A13	4	2.9	6.17	3.62E-26
ADAMTSL3	21	2.8	3.5	9.96E-08
CNTN5	15	2.8	2.54	0.00104
ESPL1	5	2.8	17.99	6.85E-47

GALNT14	11	2.8	1.13	0.0000021
KIF2C	3	2.8	12.99	5.24E-45
LOC100299874	9	2.8	1.81	6.24E-11
LOC100336690	5	2.8	4.34	1.28E-15
MKI67	26	2.8	60.13	9.44E-55
POLE	17	2.8	7.7	1.77E-28
RECQL4	14	2.8	4.06	4.54E-17
TOP2A	19	2.8	66.81	1.07E-45
UBE2C	13	2.8	20.1	1.46E-36
ZNF423	18	2.8	1.71	0.000000172
ABI3BP	1	2.7	214.81	0.0000513
BDKRB2	21	2.7	1.77	0.00000323
BMPER	4	2.7	2	0.00000013
C21H15orf42	21	2.7	4.02	2.25E-16
CASC5	10	2.7	21.43	6.38E-44
CCL26	25	2.7	2.86	0.00000384
CDCA3	5	2.7	11.33	6.83E-31
CHTF18	25	2.7	8.39	2.79E-29
FADS2	29	2.7	3.55	0.00000553
KIF15	22	2.7	6.74	2.34E-25
KIF18B	19	2.7	4.92	5.83E-19
LOC100847721	9	2.7	5.57	1.76E-19
LOC100848911	7	2.7	10.43	3.14E-33
LOC508486	25	2.7	2.31	7.15E-11
MCM4	27	2.7	12.53	5.02E-42
MCM4	14	2.7	11.77	2.45E-41
NCAPH	11	2.7	6.88	2.01E-19
NKD2	20	2.7	2.04	6.2E-10
NLRC5	18	2.7	1.98	6.78E-10
NUSAP1	10	2.7	12.27	1.96E-38
PCOLCE2	1	2.7	2.99	0.0000403
PDZD2	20	2.7	2.56	2.87E-11
PRR11	19	2.7	4.77	5.47E-17
PTPRR	5	2.7	24.11	4.26E-48
RAD54L	3	2.7	5.07	1.06E-18
SHCBP1	18	2.7	12.9	2.24E-45
TROAP	5	2.7	9.74	1.02E-32
CCNA2	6	2.6	19.32	1.53E-31
CCNB1	20	2.6	25.44	5.02E-42
CDCA7	2	2.6	3.94	9.53E-12
CENPF	16	2.6	34.69	2.15E-42
CKAP2L	11	2.6	9.6	6.81E-26
COL18A1	1	2.6	17.3	3.52E-34
FMNL1	19	2.6	4.47	6.12E-14
KIFC1	23	2.6	25.66	2.29E-46

KNTC1	17	2.6	11.44	1.03E-35
LOC512293	4	2.6	2.98	9.84E-11
MYBL2	13	2.6	29.92	2.36E-47
NEK2	16	2.6	3.81	5.58E-13
OIP5	10	2.6	3.48	1.86E-11
PLK1	25	2.6	16.39	8.75E-37
PMCH	5	2.6	1.44	0.000000589
RAD51AP1	5	2.6	2.18	9.93E-10
RASGRP2	29	2.6	4.06	2.47E-12
SPAG5	19	2.6	17.96	7.59E-38
ACVRL1	5	2.5	16.19	3.56E-17
ARHGAP11A	10	2.5	8.52	5.73E-24
BARD1	2	2.5	2.07	0.001
CDKN3	10	2.5	6.63	3.3E-19
CENPA	11	2.5	12.02	1.96E-33
CENPE	6	2.5	23.72	4.98E-45
DLGAP5	10	2.5	16.11	8.58E-33
E2F1	13	2.5	5.73	2.2E-18
ERCC6L	X	2.5	3.37	1.3E-11
ESCO2	8	2.5	5.31	2.63E-15
FBXO5	9	2.5	2.17	0.000000029
KIF11	26	2.5	29.64	5.59E-33
LMNB1	7	2.5	18.4	3.97E-30
LOC100297676	5	2.5	5.18	6.66E-15
LOC100336868	16	2.5	5.28	4.55E-13
LOC525353	26	2.5	3.15	6.33E-09
LOC786906	4	2.5	1.06	0.0000287
LPAR1	8	2.5	1.88	0.0000222
MXD3	7	2.5	2.44	2.31E-09
NAPRT1	14	2.5	6.17	9.07E-16
NCAPG2	4	2.5	19.68	1.69E-33
NDC80	24	2.5	11.52	3.47E-29
NEIL3	27	2.5	2	9.92E-09
NOD2	18	2.5	2.79	4.24E-09
PAX2	26	2.5	2.8	6.58E-10
SGOL1	1	2.5	3.88	2.11E-12
SPC25	2	2.5	3.59	1.36E-11
STMN1	2	2.5	38.27	1.78E-33
ADAMTS17	21	2.4	5.9	2.08E-12
BUB1B	10	2.4	24.35	1.44E-37
CCDC99	20	2.4	10.1	6.79E-29
CENPT	18	2.4	8.21	1.6E-18
CEP55	26	2.4	9.23	6.8E-25
CKS2	8	2.4	22.64	1.79E-34
FAM180B	15	2.4	2.52	0.0000771

GIMAP5	4	2.4	1.52	0.000166
HEYL	3	2.4	2.09	0.000000137
KIF23	10	2.4	21.84	1.33E-36
LOC782598	21	2.4	4.85	5.18E-14
NPDC1	11	2.4	6.53	7.4E-19
PDE4B	3	2.4	1.95	0.000000161
RAI2	X	2.4	2.55	0.0000205
SMC2	8	2.4	27.49	1.32E-43
SNCAIP	7	2.4	1.02	0.00155
TACC3	6	2.4	18.47	3.89E-38
TONSL	14	2.4	3.49	1.81E-11
TRIP13	20	2.4	10.57	5.67E-29
TTK	9	2.4	1.37	0.000000798
AS3MT	26	2.3	7.72	1.4E-20
AURKA	13	2.3	9.82	1.5E-24
C1QTNF1	19	2.3	45.01	1.04E-35
CCNB2	10	2.3	12.42	6.78E-27
CDCA8	3	2.3	9.82	1.86E-21
CDKN2C	3	2.3	5.16	5.06E-14
CENPN	18	2.3	7.06	7.59E-19
CORO2B	10	2.3	16.26	2.16E-26
ESM1	20	2.3	28.84	3.81E-12
FAM107B	13	2.3	1.28	0.00000857
FJX1	15	2.3	1.06	0.001
KIAA1199	21	2.3	123.5	1.24E-13
KIF20B	26	2.3	9.37	2.18E-23
LOC100848128	11	2.3	3.19	1.59E-09
LOC782601	27	2.3	1.16	0.0000362
LOC783804	26	2.3	2.17	0.000000181
MAD2L1	6	2.3	12.71	2.5E-31
MAFB	13	2.3	1.39	0.00758
MB21D1	9	2.3	1.62	0.00000537
MXRA5	X	2.3	4.05	0.0207
MYLK2	13	2.3	1.43	0.000662
NUF2	3	2.3	15.96	6.07E-26
OAF	15	2.3	13.08	6.49E-09
POLE2	10	2.3	3.26	6.82E-09
PRC1	21	2.3	42.47	3.24E-34
SCARA5	8	2.3	2.19	0.0084
SDK1	25	2.3	29.66	2.53E-19
SEMA4A	3	2.3	2.37	0.000000101
SLC43A3	15	2.3	4.31	0.000000474
UNC5C	6	2.3	16.4	2.22E-22
ANXA8L1	28	2.2	13.77	2.65E-16
AOX1	2	2.2	92.11	0.000319

BIRC5	19	2.2	23.26	8.5E-34
BUB1	11	2.2	20.12	2.36E-28
C8H9orf100	8	2.2	5.93	6.29E-14
CCDC3	13	2.2	28.74	1.01E-10
CDCA2	8	2.2	16.02	2.13E-29
CDK1	28	2.2	13.62	3.07E-28
CHAF1A	7	2.2	8.8	1.21E-14
CKAP2	12	2.2	51.02	6.41E-34
CST3	13	2.2	140.16	6.18E-15
DNA2	28	2.2	2.19	0.0000123
FAM54A	9	2.2	7.32	2.21E-18
FAM72A	16	2.2	2.74	0.000000301
FANCD2	22	2.2	12.41	1.6E-22
FGF10	20	2.2	4.31	0.000035
FKBP5	23	2.2	11.38	1.96E-25
FOSL1	29	2.2	9.17	1.87E-20
GINS2	18	2.2	5.23	9.22E-13
GPX3	7	2.2	9.08	8.89E-12
GTSE1	5	2.2	12.81	4.31E-29
IL36A	11	2.2	1.32	0.0000291
KCNE4	2	2.2	25.54	2.69E-10
KIF22	25	2.2	17.83	2.87E-29
KLK10	18	2.2	1.35	0.0263
LIMCH1	6	2.2	3.22	0.00459
LOC100337435	21	2.2	6.41	2.1E-14
LOC510844	18	2.2	7.5	2.07E-11
LOC539953	16	2.2	6.26	5.19E-14
LRRC34	1	2.2	3.54	1.23E-08
MCM7	25	2.2	40.3	7.49E-34
MELK	8	2.2	9.6	1.8E-23
METTL20	5	2.2	4.16	0.00000191
MFSD7	6	2.2	9.05	2.53E-19
PARVB	5	2.2	10.61	1.01E-21
PHF19	8	2.2	16.51	5.23E-27
PTGER4	20	2.2	5.32	1E-11
RBPMS2	10	2.2	1.14	0.00012
SFRP2	17	2.2	492.72	0.00494
SKA1	24	2.2	5.61	1.48E-13
SKA3	12	2.2	10.39	5.23E-25
ABCC9	5	2.1	1.62	0.0000609
ACER2	8	2.1	1.52	0.000106
ACOX2	22	2.1	10.85	9.24E-11
AHR	4	2.1	5.48	0.000000017
BRCA2	12	2.1	6.3	8.65E-13
C19H17orf53	19	2.1	1.23	0.000404

C1QTNF7	6	2.1	2.05	0.0496
C24H18orf54	24	2.1	2.18	0.00000475
C6H4orf21	6	2.1	5.05	0.000205
CARD6	20	2.1	2.92	0.00000124
CENPK	20	2.1	3.91	0.000000256
DIAPH3	12	2.1	41.2	4.89E-30
FAM83H	14	2.1	9.18	1.46E-09
FEN1	29	2.1	5.6	7.12E-12
GEN1	11	2.1	3.76	2.46E-08
HMMR	7	2.1	27.11	3.51E-27
ITGBL1	12	2.1	12.35	0.0000674
KIF18A	15	2.1	3.57	2.45E-08
KIF4A	X	2.1	16.82	6.25E-25
LOC100138767	18	2.1	2.18	0.0000172
LOC100301478	X	2.1	4.4	8.49E-09
LOC100848256	7	2.1	2.73	0.00000252
LOC506831	14	2.1	8	7.79E-14
MAD2	6	2.1	5.87	3.54E-12
MCM10	13	2.1	16.84	2.51E-23
MIS18BP1	21	2.1	11.62	6.51E-23
MYOC	16	2.1	6.29	4.88E-10
OGN	8	2.1	197.96	2.44E-11
PCNA	13	2.1	31.24	1.42E-25
PIK3R1	20	2.1	58.55	1.94E-25
PKD2L1	26	2.1	3.77	7.89E-08
PKIA	14	2.1	2.83	0.000953
POLA2	29	2.1	5.3	4.17E-10
REEP1	11	2.1	2.02	0.0107
REEP6	7	2.1	1.15	0.000464
TLR4	8	2.1	1.69	0.0046
TPD52	14	2.1	10.79	2.21E-15
TPX2	13	2.1	49.06	4.49E-32
TRAIP	22	2.1	1.43	0.000355
ABTB2	15	2	3.57	0.000000126
BRCA1	19	2	10.23	2.61E-20
CEP72	20	2	2.97	0.00000439
FADS1	29	2	16.48	1.89E-11
GJC1	19	2	1.25	0.000538
HMGB2	8	2	16.72	6.16E-23
HPGD	8	2	27.91	7.61E-19
LOC100847448	20	2	95.3	4.34E-24
LOC618297	4	2	13.91	1.34E-25
PAK6	10	2	2.38	0.00000892
PCDHB4	7	2	1.09	0.0182
POLQ	1	2	4.77	1.36E-09

SPRY4	7	2	1.94	0.000364
TRPC4	12	2	7.33	7E-12
WHSC1	6	2	25.5	1.03E-25
ASPA	19	-2	2.95	0.0000921
ATP8B1	24	-2	15.42	4.7E-16
CACNA1H	25	-2	24.18	0.00000121
COPG2	4	-2	33.37	2.75E-21
CPXM2	26	-2	1.26	0.00279
CRABP2	3	-2	69.14	1.6E-09
DUSP10	16	-2	4.61	0.000000311
KIAA0408	9	-2	10.95	3.56E-18
LOC100296463	25	-2	29.51	1.1E-19
LOC100335396	3	-2	2.62	0.00106
NPR3	20	-2	26.34	3.04E-19
PIK3CD	16	-2	1.58	0.000176
TBXA2R	7	-2	9.24	7.47E-16
TMEM132D	17	-2	27.17	7.29E-28
ADAMTS14	28	-2.1	3.04	0.00000077
AMY2B	3	-2.1	4.31	2.97E-08
DRAM1	5	-2.1	9.1	1.68E-14
GLIS1	3	-2.1	1.55	0.0373
GPR63	9	-2.1	7.68	4.53E-13
ITGA9	22	-2.1	4.91	7.97E-09
JAKMIP2	7	-2.1	1.39	0.00392
KLHL13	X	-2.1	5.05	8.22E-09
LMO4	3	-2.1	29.45	6.52E-08
LOC100295656	19	-2.1	1.63	0.000162
LOC516576	16	-2.1	3.91	0.000248
LOC786974	20	-2.1	16.44	0.00406
LRIG3	5	-2.1	45.54	4.3E-31
MDK	15	-2.1	2.46	0.00000256
RNF150	17	-2.1	10.1	2.37E-20
RPL7A	11	-2.1	455.5	0.00397
CCDC158	6	-2.2	1	0.00049
IL1RAP	1	-2.2	2.29	0.000000309
ITGA6	2	-2.2	9.4	6.78E-20
KCTD12	12	-2.2	6.64	1.35E-10
LOC100140687	8	-2.2	1.74	0.00000444
LOC786977	3	-2.2	4.56	0.0000271
MARCKS	9	-2.2	17.86	4.59E-17
MGC148692	6	-2.2	2.2	0.0000485
PCSK5	8	-2.2	27.04	1.81E-14
PIK3AP1	26	-2.2	5.5	2.61E-12
PTGFR	3	-2.2	4.78	6.11E-08
SCN3A	2	-2.2	4.06	0.00215

SOBP	9	-2.2	1.9	0.00000616
VWA5A	29	-2.2	5.42	0.0000513
WNT11	15	-2.2	1.49	0.00627
WNT9A	7	-2.2	1.04	0.0128
AQP1	4	-2.3	287.42	2.37E-12
CA11	18	-2.3	1.58	0.0000023
ELOVL7	20	-2.3	41.05	1.01E-29
FNBP1L	3	-2.3	5.7	2.15E-12
FOS	10	-2.3	17.17	4.4E-29
FZD1	4	-2.3	98.66	1.27E-35
GLRB	17	-2.3	6.85	0.000183
LOC511531	3	-2.3	1.19	0.0239
MYO3B	2	-2.3	1.57	0.000337
PALM3	7	-2.3	2.02	0.000341
SLC2A3	5	-2.3	35.18	2.31E-29
SLC7A8	10	-2.3	1.82	0.00000033
SPHK1	19	-2.3	16.87	0.00000206
STK31	4	-2.3	1.26	0.000332
WNT2	4	-2.3	8.22	4.4E-17
C9H6orf174	9	-2.4	4.21	8.38E-12
CILP2	7	-2.4	1.44	0.00000886
CYP7B1	14	-2.4	1.02	0.000177
FABP3	2	-2.4	37.11	2.48E-11
GDA	8	-2.4	1.52	0.0000031
GNG7	7	-2.4	7.17	3E-19
HBEGF	7	-2.4	1.04	0.0000353
IGFBP5	2	-2.4	43.9	9.79E-12
KCNJ5	29	-2.4	5.9	0.0347
PDLIM2	8	-2.4	90.16	5.14E-33
RASL11B	6	-2.4	4.36	1.86E-12
SMARCA1	X	-2.4	82.61	9.99E-49
COL8A2	3	-2.5	7.83	4.07E-16
DPYSL3	7	-2.5	368.12	1.78E-30
FAM149A	27	-2.5	2.06	0.00187
GRAMD1B	15	-2.5	1.17	0.000483
JAM2	1	-2.5	3.45	3.11E-10
NOG	19	-2.5	2.71	3.8E-10
RSPH10B	25	-2.5	1.05	0.0122
SRL	25	-2.5	4.24	1.87E-13
TBX21	19	-2.5	1.09	0.000165
TRAF5	16	-2.5	9.26	1.15E-28
ACTG2	11	-2.6	198.34	1.11E-35
ADRB2	7	-2.6	2.53	8.31E-09
C10H5orf13	10	-2.6	60.87	1.02E-26
CDH6	20	-2.6	1.06	0.0335

FHOD3	24	-2.6	3.96	2.6E-09
HTR2A	12	-2.6	1.9	0.000000379
LOC100847777	9	-2.6	1.6	0.000000209
LOC100848636	5	-2.6	2.18	0.00158
MAP1LC3C	16	-2.6	3.38	1.86E-12
PLCXD2	1	-2.6	1.76	9.82E-09
SH3GL3	21	-2.6	1.02	0.000523
TMTC2	5	-2.6	45.61	5.64E-51
B3GALNT1	1	-2.7	2.38	9.2E-11
CDO1	10	-2.7	3.21	1.29E-08
HS2ST1	3	-2.7	2.38	0.00011
LOC512486	3	-2.7	3.67	0.000209
NBEA	12	-2.7	3.95	8.93E-14
SEMA3E	4	-2.7	6.57	9.91E-24
FAT3	29	-2.8	2.28	6.87E-10
LOC100336258	17	-2.8	2.68	0.0000606
MREG	2	-2.8	6.74	7.19E-20
NEURL1B	20	-2.8	1.79	0.00000816
NTN1	19	-2.8	14.32	3E-17
P4HA3	15	-2.8	17.67	2.8E-39
PNPLA1	23	-2.8	1.59	0.000000645
TRPV2	19	-2.8	1.56	3.92E-09
CHN1	2	-2.9	61.5	4.98E-49
DLL1	9	-2.9	1.24	1.39E-08
IGDCC4	10	-2.9	2.45	1.79E-10
LOC100848933	20	-2.9	2.11	1.17E-08
RGS17	9	-2.9	9.69	5.16E-22
FAM171B	2	-3	6.43	3.96E-21
LOC100138964	20	-3	1.19	1.53E-08
LRP1B	2	-3	12.8	0.0239
RSPO2	14	-3	2.13	2.84E-08
GABRE	X	-3.1	3.17	1.96E-12
LMTK3	18	-3.1	3.16	1.51E-14
LOC100847497	X	-3.1	1.3	1.17E-09
MATN3	11	-3.1	22.56	5.17E-40
TNFSF4	16	-3.1	1.51	0.000000177
EDNRA	17	-3.2	8.17	3.24E-20
INHBA	4	-3.2	25.97	5.65E-50
SORBS2	27	-3.2	2.09	4.92E-08
GNAI1	4	-3.3	6.26	0.000000454
LOC787103	9	-3.3	6.17	6.97E-26
SRPX2	X	-3.3	9.54	7.88E-35
GLUL	16	-3.4	206.15	5.52E-60
GZMA	20	-3.4	1.33	2.01E-08
LGI2	6	-3.4	1.99	0.0000649

NKX2-2	13	-3.4	2.65	1.09E-16
STAC2	19	-3.4	1.4	9.35E-10
ABCA1	8	-3.5	3.47	0.00000113
ACTC1	10	-3.5	3.36	1.27E-17
GLULP	2	-3.5	12.45	7.08E-55
C19H17orf61	19	-3.6	12.14	7E-69
IGF2BP3	4	-3.6	9.25	2.36E-47
LOC100299139	24	-3.7	8.96	2.69E-52
THBD	13	-3.7	7.31	1.42E-39
WNT2B	3	-3.7	1.34	0.000000181
ARHGEF26	1	-3.8	2.61	7.5E-20
GDAP1L1	13	-4	2.72	6.04E-11
MUM1L1	X	-4	1.35	1.14E-10
DPT	16	-4.1	12.2	3.49E-62
ITGA8	13	-4.1	10.27	4.7E-40
TARSL2	21	-4.1	1.17	5.83E-11
COLEC12	24	-4.3	1.52	0.000000492
FEZ1	29	-4.3	2.11	1.58E-12
IGF2BP2	1	-4.3	6.92	5.59E-30
LOC522631	24	-4.3	1.39	3.89E-15
MEGF6	16	-4.4	14.5	9.74E-43
LOC100848103	25	-4.5	6.2	1.1E-26
RIMS1	9	-4.5	1.16	0.00669
TMEM40	22	-4.5	2.77	6.4E-25
TRPS1	14	-4.5	3.03	1.52E-16
LOC100848949	1	-4.6	4.49	6.01E-26
MAP2K6	19	-4.6	3.74	1.33E-13
HPCAL4	3	-4.7	2.02	0.000000036
PTHLH	5	-4.8	3.22	2.6E-11
F2R	10	-5.1	1.28	1.28E-17
LOC517509	10	-5.2	4.35	9.73E-13
LTBP1	11	-5.2	92.68	2.66E-119
SPOCK1	7	-5.2	6.42	4.61E-21
MEST	4	-5.3	10.86	6.07E-75
SEMA3D	4	-5.3	39.26	5.59E-49
ATP6AP1L	7	-5.4	8.7	1.88E-45
SRSF12	9	-5.5	1.26	3.04E-15
LOC100847414	4	-5.6	30.31	1.43E-32
PGM5	8	-5.7	6.4	7.2E-63
CTNNA2	11	-6	6.94	6.06E-63
COL2A1	5	-6.1	1.79	0.0382
DFNA5	4	-6.2	1.05	3.08E-17
POSTN	12	-6.7	149.69	2.86E-23
BRSK1	18	-7.2	1.41	3.71E-25
RUNX3	2	-7.8	1.16	6.91E-13

LOC100848767	4	-13.7	3.93	4.74E-60
LOC784924	4	-14.4	1.98	1.91E-30
EYA4	9	-18.1	8.63	1.13E-83
CD200R1L	1	-20.8	6.47	2.42E-20

Hour 2

**Gene**	**Chromosome**	**Fold Change**	**CPM**	**FDR**
PTX3	1	67.7	5.27	9.1E-18
ACAN	21	45.8	12.59	1.07E-12
NTS	5	34.6	3.29	1.07E-12
DTX4	15	32.2	3.06	1.94E-12
GREB1	11	31.1	1.76	0.00000202
CCL20	2	18.1	2.15	0.00000486
TBX2	19	17	2.28	5.37E-08
C2CD4B	10	15.4	2.66	0.000000119
RND1	5	14.4	71.34	1.17E-10
MAN1C1	2	13.5	1.88	0.00000784
LOC532218	5	13	38.61	6.69E-09
SLCO4A1	13	13	2.36	0.000000171
S1PR1	3	12.6	1	0.00000109
ARHGDIB	5	12.2	1.23	0.0000139
LOC100847493	20	11.9	1.61	0.000000294
CX3CL1	18	11.6	3.6	9.47E-08
COL8A1	1	11.1	7.88	0.00000828
FGD5	22	10.9	5.12	0.000000185
CCL5	19	10.7	55.58	0.0000229
IL6	4	10.5	20.73	1.85E-08
CLDN11	1	10.4	1.6	0.00000683
TNF	23	10.4	4.15	0.000000346
CFB	23	10.2	12.64	0.000000292
IFI27	21	10.2	1.25	0.0000164
PTGIS	13	9.6	35.82	0.000000105
LOC100847495	23	9.4	4.99	0.00000151
MGLL	22	9.2	23.76	7.91E-08
RARRES1	1	9.2	57.63	0.000000283
LOC509420	8	8.7	1.01	0.0000451
FES	21	8.4	9.16	0.00000135
MASP1	1	8.1	10.15	0.0000242
CA13	14	7.7	3.56	0.000486
CD83	23	7.7	7.12	0.00000307
KCNMB1	20	7.6	2.29	0.0332
LOC100847896	15	7.6	1.47	0.00018
LOC516579	13	7.5	1.97	0.000127
MT2A	18	7.5	24.32	0.00995
TNN	16	7.5	2.52	0.0043

CCL2	19	7.3	296.65	0.00000245
BCAS1	13	7.2	2.91	0.0000336
MN1	17	7.2	2.26	0.00529
LOC618369	5	7.1	13.15	0.00000471
CACNA1C	5	6.9	2.44	0.000154
KLK10	18	6.9	3.34	0.0000811
LOC789200	29	6.9	3.06	0.011
MOCOS	24	6.6	2.84	0.00274
CDA	2	6.5	2.34	0.000167
LSP1	29	6.5	10.88	0.000261
SESN3	15	6.5	11.72	0.0282
TFAP2A	23	6.5	3.08	0.000196
AFAP1L2	26	6.4	9.4	0.000218
LOC100847561	23	6.2	1.2	0.000516
ELMO3	18	6	1.19	0.00266
GPR132	21	5.8	5.38	0.000461
IL8	6	5.8	33.63	0.000135
SDK2	19	5.8	1.89	0.000974
TSTD1	3	5.7	5	0.00198
SEMA5A	20	5.6	5.32	0.000768
LOC616198	9	5.5	1.85	0.00104
PTGS2	16	5.5	118.09	0.00353
TCF19	23	5.5	3.53	0.00551
CRTAC1	26	5.4	1.99	0.00327
CCL26	25	5.3	4.97	0.00199
FADS2	29	5.3	3.4	0.00507
HCN4	10	5.2	2.52	0.00264
LOC100138376	16	5.2	71.18	0.00893
RPS6KA1	2	5.2	1.35	0.00497
TOX	14	5.2	3.46	0.0297
HSD17B14	18	5.1	3.57	0.0307
SMOC2	9	5.1	43.11	0.00361
BDKRB1	21	5	3.13	0.00507
CPZ	6	5	4.19	0.00243
ELN	25	5	387.35	0.00171
IER3	23	5	469.59	0.00292
CXCL2	6	4.9	177.21	0.000222
N4BP3	7	4.9	11.61	0.00276
RAD54L	3	4.9	4.15	0.00651
TRIM47	19	4.8	16.54	0.00559
EGLN3	21	4.7	8.78	0.00112
CDT1	18	4.6	2.82	0.0245
GALNT14	11	4.6	1.06	0.0319
HAS2	14	4.6	6.84	0.0413
HHIPL1	21	4.6	8.41	0.00737

C28H10orf10	28	4.5	2.02	0.0223
MCM5	5	4.5	21.4	0.00538
TNIP1	7	4.5	152.86	0.00274
CRYAB	15	4.4	56.95	0.0092
CDCA3	5	4.3	14.97	0.0183
LOC100337435	21	4.3	43.14	0.0118
NFKBID	18	4.3	9.72	0.00422
CDC20	3	4.2	23.55	0.0118
GSTO2	26	4.2	1.12	0.0173
PDE4B	3	4.2	7.84	0.00476
PERP	9	4.2	5.02	0.011
CARD11	25	4.1	3.31	0.021
CD40	13	4	12.33	0.0053
CDCA5	29	4	2.6	0.0232
CYP1A1	21	4	5.87	0.0133
GPX3	7	4	15.69	0.00384
NAPRT1	14	4	8.92	0.0359
NFATC1	24	4	126.68	0.00436
TLR2	17	4	1	0.0341
CD34	16	3.9	3.71	0.0228
IL1RL1	11	3.9	18.65	0.00991
NKD2	20	3.9	3.53	0.0356
LOC100848911	7	3.8	12.12	0.0308
LOC790886	16	3.8	6.66	0.0156
C15H11orf96	15	3.7	114.03	0.0109
MAP3K8	13	3.7	11.68	0.019
TROAP	5	3.7	6.22	0.0166
MCM4	14	3.6	11.81	0.0297
UBE2C	13	3.5	21.49	0.0223
RRM2	11	3.4	32.4	0.0242
STMN1	2	3.3	37.87	0.0243
UHRF1	7	3.3	10.91	0.032
MCM3	23	3.2	14.49	0.0459
LOC100848038	7	3.1	228.65	0.0287
RCAN1	1	3	72.58	0.0459
CDKN2B	8	-3.4	33.76	0.0352
CHN1	2	-3.6	51.68	0.0232
LRIG3	5	-3.6	10.05	0.0458
STAC2	19	-3.9	1.43	0.0386
MAP2K6	19	-4.3	3.34	0.00624
FAT3	29	-4.4	1.69	0.0142
SRSF12	9	-4.4	1.01	0.0234
IGF2BP2	1	-4.5	6.05	0.00624
COL8A2	3	-4.6	7.99	0.0491
GNAI1	4	-4.6	5.34	0.0447

THBD	13	-4.6	9.98	0.00127
ADRB2	7	-4.7	1.33	0.0254
ATP6AP1L	7	-4.7	9.33	0.00289
KCNJ5	29	-4.9	4.72	0.00476
IGF2BP3	4	-5	6.92	0.0024
MATN3	11	-5	18.88	0.038
PGM5	8	-5.2	5.56	0.000612
ITGA8	13	-5.5	8.9	0.00134
TRPS1	14	-6	2.4	0.00101
SPOCK1	7	-6.2	5.77	0.00139
CTNNA2	11	-6.4	6.44	0.0000752
MEGF6	16	-6.5	12.58	0.0000218
MEST	4	-6.5	9.13	0.0000881
ARHGEF26	1	-6.6	1.81	0.000974
LTBP1	11	-7	81.68	0.0000981
RUNX3	2	-7.2	1	0.000946
MREG	2	-7.5	5.24	0.0198
LOC100847414	4	-7.9	26.32	0.000222
SEMA3D	4	-9.5	34.57	0.00127
POSTN	12	-9.6	130.43	0.0000298
MUM1L1	X	-9.8	1.21	0.0352
LOC100848767	4	-11.5	3.01	0.00000151
LOC784924	4	-11.7	1.7	0.00000023
BRSK1	18	-18	1.2	0.00000246
CD200R1L	1	-23.6	5.24	0.00000216
EYA4	9	-28.1	8.85	1.94E-09

Hour 8

Gene	Chromosome	Fold Change	CPM	FDR
NPY1R	6	94.5	0.82	3.45E-66
TMEM100	19	59.2	0.8	3.41E-57
GREB1	11	48.7	1.18	1.19E-72
MX2	1	33.9	1.04	8.52E-46
MMP3	15	27.8	0.16	3.46E-39
NTS	5	27.7	2.21	6.57E-91
DTX4	15	20.7	1.25	1.45E-33
TGM3	13	20.4	3.77	4.31E-58
ACAN	21	19	4.06	5.42E-64
MAN1C1	2	18.8	2.06	9.89E-28
OAS1	17	12.8	5.1	1.08E-28
COL8A1	1	10.9	2.87	1.41E-51
OAS2	17	10.6	2.23	2.05E-28
LOC516579	13	10.3	0.43	3.91E-16
CCL20	2	10.1	0.48	6.52E-21
S1PR1	3	9.8	0.04	3.48E-23

MASP1	1	9.3	3.78	1.14E-19
MAOA	X	9.2	0.69	1.04E-15
BCAS1	13	8.6	1.95	6.99E-36
MN1	17	8.5	0.85	4.43E-30
HRH1	22	8.4	0.21	2.19E-19
FGD5	22	8.2	1.6	3.41E-38
IL6	4	7.9	5.23	1.36E-34
FES	21	7.7	3.44	1.59E-98
LOC782202	3	7.7	1.94	0.00818
RARRES1	1	7.6	5.62	1.99E-63
LOC100847495	23	7.5	1.61	2.45E-35
AFAP1L2	26	7.4	2.56	5.81E-60
IFI27	21	7.3	1.99	8.32E-39
SDK2	19	7.1	1.94	2.28E-39
SESN3	15	7.1	2.62	6.21E-24
CDC6	19	6.9	2.3	8E-16
CLDN11	1	6.9	1.45	8.85E-29
PDPN	16	6.8	1.09	0.0000586
PAQR7	2	6.7	0.27	1.39E-22
CA13	14	6.4	0.99	1.92E-10
E2F8	29	6.4	1.61	1.16E-15
SEMA5A	20	6.3	2.45	1.87E-48
CACNA1C	5	6	1.32	2.65E-28
BDKRB1	21	5.9	2.98	3.03E-32
TFAP2A	23	5.9	2.9	8.56E-58
TOX	14	5.9	1.55	2.2E-30
TBX2	19	5.8	0.05	1.96E-17
ITPR3	23	5.7	0.48	6.55E-15
PTX3	1	5.7	2.32	5.05E-25
DRD3	1	5.5	0.62	1.22E-20
HAS2	14	5.5	2.86	4.58E-44
CYP1A1	21	5.4	2.19	1.55E-25
SAA3	29	5.1	1.53	8.65E-09
SMOC2	9	5.1	5.34	6.66E-14
KCNMB1	20	5	1.43	1.49E-22
STRA6	21	5	0.92	2.82E-10
TNN	16	5	1.47	8.24E-12
CRTAC1	26	4.9	1.73	3.11E-10
DACT2	9	4.9	2.04	2.19E-22
PTGIS	13	4.9	4.97	1.02E-94
FOXM1	5	4.7	1.09	5.71E-17
LOC618369	5	4.7	2.92	7.17E-32
GPR132	21	4.6	0.17	3.53E-15
KLK10	18	4.6	1.64	0.000000342
MGLL	22	4.5	3.81	4.78E-37

ISG15	16	4.4	2.38	0.000000344
KITLG	5	4.4	1.84	3.54E-19
LOC512286	3	4.4	0.03	1.77E-12
HJURP	3	4.3	3.07	6.8E-41
HSD17B14	18	4.3	0.8	5.96E-09
MOCOS	24	4.3	2.4	2.64E-26
EXO1	16	4.2	1.55	0.000000448
MX1	1	4.2	4.82	2.67E-19
OLFML1	15	4.2	1.64	0.0016
CDA	2	4.1	1.3	1.72E-10
CDC25B	13	4.1	4.22	6.31E-85
FAM83D	13	4.1	1.74	1.2E-09
LOC100299874	9	4.1	0.05	3.62E-12
PDE4B	3	4.1	3.92	2.79E-43
COL15A1	8	4	7.8	2.95E-34
FADS2	29	4	1.53	2.48E-12
LOC100297676	5	4	4.74	8.94E-74
MXD3	7	4	0.08	3E-11
EGLN3	21	3.9	3.3	1.03E-37
RRM2	11	3.9	5.95	2.56E-43
C2	23	3.8	1.17	0.00000121
CAMK1G	16	3.8	0.19	0.00000929
CDCA5	29	3.8	1.93	7.34E-15
CPZ	6	3.8	1.81	8.95E-16
ELN	25	3.8	8.41	1.53E-12
NEK2	16	3.8	1.31	3.9E-18
UBE2C	13	3.8	3.66	2.99E-46
CDC20	3	3.7	3.91	1.92E-64
CFB	23	3.7	6.4	1.17E-60
CMPK2	11	3.7	1.44	3.83E-09
FAM64A	19	3.7	3	2.78E-24
GALNT14	11	3.7	0.51	1.86E-08
IL1RL1	11	3.7	5.39	7.65E-25
KIF18B	19	3.7	2.01	9.32E-20
TOP2A	19	3.7	5.44	3.71E-59
TROAP	5	3.7	2.7	1.21E-26
CCNF	25	3.6	2.73	3.8E-22
CD34	16	3.6	1.96	2.82E-11
FMNL1	19	3.6	1.73	1.38E-08
KCNMA1	28	3.6	2.43	8.25E-19
KIF2C	3	3.6	3.48	5.36E-32
MKI67	26	3.6	5.31	3.69E-69
UHRF1	7	3.6	4.33	3.85E-45
BDKRB2	21	3.5	2.29	3.18E-17
FBXO5	9	3.5	1.1	1.73E-12

KIAA0101	10	3.5	2.7	1.21E-26
MARVELD2	20	3.5	0.42	3.49E-11
MXRA5	X	3.5	3.96	9.77E-09
NEIL3	27	3.5	0.95	5.03E-09
SOX13	16	3.5	0.71	0.0000228
SP100	2	3.5	1.09	0.000000803
SPAG5	19	3.5	3.55	2.17E-36
ASPM	16	3.4	4.13	6.1E-46
CCL5	19	3.4	5.47	1.27E-25
CLSPN	3	3.4	2.38	8.44E-19
FAM49A	11	3.4	0.72	2.32E-10
GFPT2	7	3.4	7.7	3.75E-39
HEYL	3	3.4	1.29	8.84E-14
LOC512293	4	3.4	1.12	1.84E-15
LSP1	29	3.4	2.21	4.09E-16
MT2A	18	3.4	3.84	7.33E-16
PTGS1	11	3.4	5.75	9.81E-12
ASF1B	7	3.3	2.2	9.77E-16
BST2	7	3.3	4.67	1.12E-12
CCNB1	20	3.3	4.14	1.14E-57
CDCA3	5	3.3	2.57	8.01E-23
CDT1	18	3.3	2.33	5.3E-13
CENPA	11	3.3	3.1	7.49E-27
IL8	6	3.3	6.02	8.28E-45
KIF20A	7	3.3	4.73	1.24E-40
NOD2	18	3.3	2.61	4.72E-18
OLR1	5	3.3	4.95	1.9E-32
PERP	9	3.3	2.61	2.34E-22
RND1	5	3.3	3.53	8.35E-20
TFAP4	25	3.3	0.48	0.00000387
TSTD1	3	3.3	1.42	3.3E-14
CCL2	19	3.2	7.88	3.78E-26
CCNA2	6	3.2	3.75	5.34E-32
ESPL1	5	3.2	3.74	2.2E-40
KIF15	22	3.2	2.52	1.58E-22
LOC100848911	7	3.2	2.86	6.35E-24
ADAMTS16	20	3.1	0.65	2.3E-09
CENPF	16	3.1	4.26	6.86E-46
COL18A1	1	3.1	4.27	1.86E-52
DEPDC1	3	3.1	2.34	9.68E-20
ERCC6L	X	3.1	1.68	3.49E-14
IQGAP3	3	3.1	3.52	5.59E-30
LOC100848019	9	3.1	5.78	2.44E-12
LOC100848300	18	3.1	1.23	5.41E-08
LOC790886	16	3.1	2.41	3.41E-15

PRR11	19	3.1	1.74	5.7E-15
PRUNE2	8	3.1	4.34	9.42E-37
SLC4A4	6	3.1	4.33	2.05E-48
STMN1	2	3.1	4.52	7.99E-59
C21H15orf42	21	3	2.21	1.32E-16
CCL26	25	3	1.07	3.28E-08
CDCA7	2	3	3.15	1.95E-16
CFH	16	3	5.74	3.26E-36
DAAM2	23	3	3.36	2.09E-13
EFR3B	11	3	0.21	0.00509
ESCO2	8	3	2.22	3.24E-18
LMNB1	7	3	4.42	3.99E-41
LOC790886	16	3	1.15	9.63E-12
NUSAP1	10	3	2.88	9.94E-22
PTGS2	16	3	5	3.15E-51
PTPRB	5	3	0.65	4.97E-09
SLC25A13	4	3	2.58	1.97E-21
SLFN11	19	3	3.27	7.79E-11
SOCS2	5	3	2.45	9.17E-19
CCNB2	10	2.9	2.95	4.23E-22
CORO2B	10	2.9	3.32	2.19E-23
CX3CL1	18	2.9	0.26	0.0117
CYP3A4	25	2.9	5.52	3.17E-13
F13A1	23	2.9	9.02	7.93E-22
HHIPL1	21	2.9	2.75	4.94E-22
LOC100138376	16	2.9	4.3	1.89E-48
LOC100298822	23	2.9	1.92	1.12E-09
LOC100336368	4	2.9	1.55	2.42E-11
LOC100847802	25	2.9	0.23	0.0000756
LOC781004	16	2.9	1.96	4E-13
NCAPH	11	2.9	2.49	7.75E-18
SGOL1	1	2.9	1.83	3.48E-14
SHCBP1	18	2.9	3.28	7.26E-21
ABI3BP	1	2.8	7.8	0.00000329
ARHGAP11A	10	2.8	2.79	7.91E-20
CA4	19	2.8	1.66	0.00271
CARD11	25	2.8	2.09	5.81E-12
CASC5	10	2.8	3.95	9.46E-42
CENPT	18	2.8	2.57	5.33E-17
ITGA7	5	2.8	3.11	0.0000051
KCNE4	2	2.8	4.86	2.22E-25
KNTC1	17	2.8	3.38	2.19E-24
LOC100336535	19	2.8	0.82	2.69E-08
MCM5	5	2.8	4.75	2.07E-23
MYB	9	2.8	0.39	0.000000349

MYBL1	14	2.8	1.86	1.02E-08
MYBL2	13	2.8	4.47	5.83E-38
NDC80	24	2.8	2.83	2.07E-22
PAX2	26	2.8	1.17	4.08E-09
PCOLCE2	1	2.8	1.86	0.000476
PLK1	25	2.8	3.38	1.72E-26
POLE	17	2.8	3.26	1.14E-18
RAD51AP1	5	2.8	1.5	1.87E-09
RAD54L	3	2.8	2.77	2.14E-18
SOD2	9	2.8	3.74	1.26E-10
TCF19	23	2.8	2.08	2.81E-13
ZBP1	13	2.8	0.96	2.36E-08
ABTB2	15	2.7	1.42	9.48E-10
BUB1B	10	2.7	4.02	6.21E-37
DHX58	19	2.7	2.44	6.75E-09
DLGAP5	10	2.7	3.48	8.3E-25
FAM105A	20	2.7	1.49	0.00000306
GPX3	7	2.7	3.71	1.38E-21
IL1RL2	11	2.7	0.49	0.00119
KIF11	26	2.7	4.47	6.86E-46
LOC100847721	9	2.7	2.05	1.53E-11
LOC507055	3	2.7	0.53	0.00027
LOC508486	25	2.7	0.51	0.000000126
MAPK12	5	2.7	1.5	3.65E-08
MB21D1	9	2.7	1.84	9.06E-10
MCM3	23	2.7	4.94	1.02E-23
OIP5	10	2.7	1.51	9.19E-12
PHF21B	5	2.7	1.04	1.03E-09
PLCE1	26	2.7	0.74	1.99E-08
PTPN5	29	2.7	0.54	0.0000107
TM6SF2	7	2.7	0.24	0.0213
ASB9	X	2.6	0.1	0.0000556
C1QTNF1	19	2.6	5.67	5.55E-31
CCBE1	24	2.6	3.09	0.000000837
CDCA2	8	2.6	3.68	5.04E-31
CENPE	6	2.6	4.43	1.23E-40
CHAF1A	7	2.6	3.73	9.42E-23
CKS2	8	2.6	3.86	1.65E-34
CRYAB	15	2.6	4.89	4.69E-33
FANCD2	22	2.6	3.43	2.05E-21
GSTT4	17	2.6	0.6	0.0000247
IRF7	29	2.6	3.09	1.81E-20
LOC100336868	16	2.6	2.44	1.51E-14
LOC525353	26	2.6	1.74	1.7E-11
LOC539953	16	2.6	3.74	7.06E-25

PRSS48	17	2.6	0.22	0.0000199
PTPN3	8	2.6	2.04	1.66E-12
PTPRD	8	2.6	2.87	1.22E-12
TLR2	17	2.6	1.42	0.000897
TLR4	8	2.6	0.71	0.0000135
ACSL5	26	2.5	6.81	1.72E-30
BIRC5	19	2.5	4.09	3.06E-32
C19H17orf53	19	2.5	0.3	0.0000215
C8H9orf100	8	2.5	2.15	5.47E-12
CDK1	28	2.5	3.43	7.8E-22
CEP55	26	2.5	2.87	2.15E-15
CHTF18	25	2.5	2.69	2.18E-15
GPRC5A	5	2.5	0.53	0.0000205
KIF23	10	2.5	4.08	4.91E-36
LOC100336690	5	2.5	2.32	1.96E-11
LOC614091	23	2.5	1.76	0.00403
LPAR1	8	2.5	0.91	0.000445
MCM2	22	2.5	4.75	1.32E-20
MCM4	27	2.5	4.1	2.62E-20
MCM4	14	2.5	4.02	4.19E-20
NR4A2	2	2.5	2.98	7.01E-16
TACC3	6	2.5	3.82	4.13E-22
ALOX12	19	2.4	0.53	0.000832
ANXA8L1	28	2.4	4.31	7.6E-23
AQPEP	10	2.4	0.87	0.00000168
ATAD5	19	2.4	2.62	6.36E-14
BNC2	8	2.4	3.33	4.93E-18
BUB1	11	2.4	3.66	5.88E-25
CDKN3	10	2.4	2.15	3.29E-09
CHD7	14	2.4	1.46	0.000042
CKAP2L	11	2.4	3.04	2.84E-18
DNA2	28	2.4	1.35	0.000000294
FEN1	29	2.4	3.04	1.34E-13
ID1	13	2.4	4.97	3.19E-35
KIFC1	23	2.4	4.01	4.94E-24
MCM8	13	2.4	1.04	0.000336
MEOX2	4	2.4	2.43	1.99E-08
MFSD7	6	2.4	3.09	1.4E-17
PION	4	2.4	1.05	0.000216
PRC1	21	2.4	5.04	9.7E-35
SFRP2	17	2.4	8.85	0.00106
SMC2	8	2.4	4.84	5.19E-23
TBX3	17	2.4	2.4	3.56E-12
TNIP1	7	2.4	7.23	9.02E-12
ACER2	8	2.3	0.58	0.0000395

ADAMTSL3	21	2.3	1.65	0.000000445
CBFA2T3	18	2.3	3.12	1.15E-15
CCDC3	13	2.3	4.91	2.85E-13
CCDC99	20	2.3	3.31	6.06E-15
CD40	13	2.3	4.4	2.4E-18
CDCA8	3	2.3	2.85	2.8E-14
CDKN2C	3	2.3	1.74	1.84E-08
DEPDC1B	20	2.3	1.12	0.000000356
E2F1	13	2.3	2.96	8.71E-13
FADS1	29	2.3	3.78	1.17E-22
FAM111B	15	2.3	2.89	2.22E-13
FAM72A	16	2.3	1.27	0.000000109
FGF13	X	2.3	2.98	3.52E-10
FGF7	10	2.3	6.57	1.91E-26
FJX1	15	2.3	0.03	0.000118
HSD3B1	3	2.3	0.46	0.0000727
IL36A	11	2.3	2.61	1.68E-11
KIF22	25	2.3	3.71	5.03E-21
LOC100848985	4	2.3	1.36	0.0000115
MAD2L1	6	2.3	3.24	5.68E-17
MCM7	25	2.3	5.4	2.76E-20
MFSD6	2	2.3	2.98	3.45E-14
NKD2	20	2.3	1.2	0.000000301
OAF	15	2.3	3.75	3.49E-14
PLA2G4A	16	2.3	5.95	1.45E-23
RECQL4	14	2.3	2.2	1.38E-08
SKA3	12	2.3	2.92	3.31E-16
ST8SIA1	5	2.3	2.97	0.000000106
TONSL	14	2.3	2.31	5.87E-08
ACSS1	13	2.2	1.46	0.0139
ADAMTS17	21	2.2	2.39	9.1E-09
ADAMTS5	1	2.2	4.52	8.83E-18
BRCA1	19	2.2	3.43	2.71E-16
CA2	14	2.2	6.54	4.16E-24
CENPN	18	2.2	2.42	6.33E-10
CYP1B1	11	2.2	6.37	8.94E-13
EPSTI1	12	2.2	3.58	5.81E-10
ESM1	20	2.2	6.1	3.96E-15
FAM54A	9	2.2	2.93	3.99E-12
GATM	10	2.2	1.33	0.0000564
GEN1	11	2.2	2.3	8.3E-09
GINS2	18	2.2	2.32	1.25E-08
GTSE1	5	2.2	3.6	1.25E-18
HMGB3	X	2.2	0.53	0.0000967
KIF20B	26	2.2	2.97	7.24E-15

LGALS4	18	2.2	0.04	0.000443
LIMCH1	6	2.2	1.41	0.00843
LOC510844	18	2.2	2.91	5.45E-12
LOC783804	26	2.2	0.72	0.0000141
MCM10	13	2.2	4.8	6.14E-23
MIS18BP1	21	2.2	3.19	9.77E-16
NAPRT1	14	2.2	1.78	0.000000602
NLRC5	18	2.2	1.49	0.000000916
NUF2	3	2.2	3.5	3.47E-17
ORC1	3	2.2	2.2	0.000000519
PHF19	8	2.2	3.39	3.41E-17
PKD2L1	26	2.2	1.42	0.000000157
POLA2	29	2.2	2.88	3.3E-10
PTPRQ	5	2.2	2.51	0.000625
RTP4	1	2.2	2.08	0.00000597
TRAIP	22	2.2	0.67	0.0000562
TRIP13	20	2.2	3.62	1.3E-16
VAT1L	18	2.2	3.63	0.00935
WHSC1	6	2.2	4.42	2.86E-23
ACVRL1	5	2.1	3.63	1.74E-13
ADA	13	2.1	3.7	7.67E-11
AHR	4	2.1	2.97	1.58E-10
APITD1	16	2.1	3.06	1.16E-10
ASAP2	11	2.1	1.32	0.0000102
AURKA	13	2.1	3	8.16E-12
AVIL	5	2.1	1.15	0.0000337
C10H15orf48	10	2.1	3.1	0.0000611
C24H18orf54	24	2.1	1.49	0.00000973
C3	7	2.1	3.12	0.000000174
C5H12orf48	5	2.1	2.19	1.66E-08
CD1D	3	2.1	1.3	0.000194
CDC25A	22	2.1	3.08	1.32E-09
CDC45	17	2.1	3.23	1.6E-11
CEACAM1	18	2.1	0.63	0.00517
CEP72	20	2.1	1.65	0.00000047
CKAP2	12	2.1	5.26	2.42E-26
FIGNL1	4	2.1	1.49	0.00000115
GINS4	27	2.1	1.99	0.00000035
GMNN	23	2.1	3.46	1.18E-13
HMMR	7	2.1	4.33	2.47E-25
HOMER2	21	2.1	0.99	0.0278
IFIH1	2	2.1	2.52	0.00053
IL34	18	2.1	3.02	6.21E-08
INCENP	29	2.1	3.65	9.66E-18
KIF1A	3	2.1	0.37	0.00526

MASTL	13	2.1	2.56	7.12E-10
MIR147	10	2.1	0.89	0.000198
MMP19	5	2.1	4.99	1.47E-17
MND1	17	2.1	1.51	0.00000983
MYO10	20	2.1	6.64	3.54E-20
PARVB	5	2.1	3.28	4.11E-12
PCNA	13	2.1	5.24	1.38E-24
PITPNC1	19	2.1	2.35	0.000000283
POLE2	10	2.1	2.31	2.76E-08
POLQ	1	2.1	1.97	0.000000734
RAI2	X	2.1	0.39	0.000738
RASGRP2	29	2.1	1.75	0.000000214
SKA1	24	2.1	2.51	3.92E-10
TOB1	19	2.1	4.06	5.06E-10
TPX2	13	2.1	5.37	5.53E-23
XAF1	19	2.1	0.29	0.014
ARHGAP22	28	2	0.95	0.0000935
C1QTNF3	20	2	6.38	1.06E-12
C26H10orf131	26	2	0.25	0.026
CADPS2	4	2	0.19	0.00519
CENPH	20	2	2.03	0.000000477
CXCL6	6	2	9.14	0.0000181
DDX58	8	2	4.21	2.48E-10
HERC6	6	2	4.61	1.31E-22
HHAT	16	2	0.5	0.00048
KIF18A	15	2	1.92	0.00000318
LOC100848128	11	2	1.13	0.0000217
LOC100848681	18	2	0.52	0.000813
LOC508347	3	2	1.67	0.00243
LOC782598	21	2	2.03	0.000000766
LOC788634	23	2	2.56	0.000539
PALB2	25	2	2.63	1.07E-09
PAPSS2	26	2	5.06	1.57E-23
PRG4	16	2	1.2	0.00417
PRR5L	15	2	4.39	4.32E-25
PTGDR	10	2	4.88	4.17E-11
STIL	3	2	2.32	0.000000062
TREX1	22	2	1.81	0.00000259
AHNAK2	21	-2	4.26	2.51E-15
APOE	18	-2	3.12	6.54E-12
C27H8orf48	27	-2	1.12	0.0000169
CH25H	26	-2	4.01	0.00000474
EGFR	22	-2	3.54	2.25E-11
FABP3	2	-2	4.88	4.65E-11
FAT4	17	-2	5.96	1.02E-17

KIAA0408	9	-2	3.82	1.88E-17
LMCD1	22	-2	2.82	1.23E-10
LOC535166	14	-2	4.94	0.000000207
LOC783163	13	-2	0	0.00182
MAP1LC3C	16	-2	2.43	0.00000329
NTN1	19	-2	4.05	1.09E-09
OSR1	11	-2	2.94	3.28E-10
PITX2	6	-2	0.36	0.00909
SLC9A3R2	25	-2	2.05	0.00000312
ZNF516	24	-2	0.79	0.00306
ADAMTS20	5	-2.1	4.33	1.73E-12
AMIGO2	5	-2.1	4.4	1.62E-25
ATP8B1	24	-2.1	4.06	1.55E-19
CCDC88B	29	-2.1	0.04	0.000291
CD1A	3	-2.1	1.28	0.0016
COPG2	4	-2.1	4.86	8.77E-19
DDC	4	-2.1	0.92	0.0000489
DFNA5	4	-2.1	0.62	0.000183
EGR1	7	-2.1	2.92	0.00012
GRAMD1B	15	-2.1	0.23	0.0262
HSPB8	17	-2.1	4.97	1.13E-19
JPH2	13	-2.1	3.16	9.04E-14
MDK	15	-2.1	1.13	0.00000929
NHSL1	9	-2.1	3.47	1.99E-12
PCSK1N	X	-2.1	1.07	0.000069
PEG10	4	-2.1	3.08	1.91E-12
PTK7	23	-2.1	6.63	4.75E-21
RAPGEF3	5	-2.1	1.65	0.00000431
RRAGD	9	-2.1	0.97	0.00011
TMEM132D	17	-2.1	4.82	1.38E-14
TPPP3	18	-2.1	0.86	0.0000472
WDR35	11	-2.1	5.37	4.62E-20
AKAP6	21	-2.2	4.63	1.23E-11
AMY2B	3	-2.2	1.99	0.000000088
COL7A1	22	-2.2	2.87	5.44E-10
CRABP2	3	-2.2	6.12	1.71E-15
DLL1	9	-2.2	0.11	0.00205
DSC3	24	-2.2	6.97	1.93E-18
DUSP10	16	-2.2	2.09	2.02E-09
FAM131B	4	-2.2	1.53	0.000263
FNDC1	9	-2.2	5.88	1.29E-22
FZD1	4	-2.2	6.21	9.63E-25
HAVCR2	7	-2.2	0.2	0.00431
HNMT	2	-2.2	4.75	4.58E-18
IL15	17	-2.2	2.44	1.57E-08

INHBA	4	-2.2	5.33	1.87E-25
KLHL13	X	-2.2	1.34	0.0000193
LOC100847429	13	-2.2	5.2	2.33E-33
LOC539209	18	-2.2	2.7	4.17E-11
LOC783891	9	-2.2	6.13	2.69E-22
PAK1	29	-2.2	4.64	4.61E-15
PCNXL2	28	-2.2	1.68	0.00000183
PHLDB2	1	-2.2	6.64	4.43E-21
SLCO3A1	21	-2.2	1.33	0.000025
SRPX2	X	-2.2	3.99	6.4E-21
TYRO3	10	-2.2	4.49	2.8E-29
WFDC1	18	-2.2	4.64	3.24E-10
C9H6orf174	9	-2.3	2.5	2.26E-12
DOCK10	2	-2.3	4.99	2.76E-31
FGF2	17	-2.3	5.52	4.08E-26
GIPC3	7	-2.3	0.42	0.000068
HMGA1	23	-2.3	4	3.73E-23
IGFBP5	2	-2.3	5.04	0.00000435
LOC100295656	19	-2.3	0.56	0.000359
MDFI	23	-2.3	0.65	0.000267
NUDT6	17	-2.3	4.7	2.92E-23
ODZ4	29	-2.3	4.27	7.31E-16
SCRN1	4	-2.3	2.58	5.89E-09
SLC6A9	13	-2.3	3.29	1.15E-15
FAT3	29	-2.4	0.52	0.000822
IGDCC4	10	-2.4	1.39	0.000000388
ISLR	21	-2.4	1.54	0.011
ITGA6	2	-2.4	3.21	2.81E-19
KCNJ5	29	-2.4	3.59	0.000231
LIMS2	2	-2.4	3.1	3.97E-18
LOC100848933	20	-2.4	1.74	0.00000782
MMP11	17	-2.4	1.8	7.85E-09
PIK3CD	16	-2.4	0.49	0.00003
RNF150	17	-2.4	3.44	3.4E-20
SLC16A2	X	-2.4	1.7	0.0000341
SPHK1	19	-2.4	4.13	9.13E-14
ADAMTSL1	8	-2.5	2.26	0.0000012
BEND6	23	-2.5	0.42	0.036
C10H5orf13	10	-2.5	6.02	8.85E-24
COLEC12	24	-2.5	0.62	0.0000344
FHL1	X	-2.5	4.16	2.68E-39
FNBP1L	3	-2.5	2.7	4.32E-14
FNDC4	11	-2.5	1.98	5.39E-11
ITGA9	22	-2.5	2.41	5.63E-13
NBEA	12	-2.5	2	5.68E-10

TMEM8B	8	-2.5	0.57	0.00863
ADAMTSL1	8	-2.6	4.03	8.39E-13
C1QTNF6	5	-2.6	3.35	6.09E-21
CA11	18	-2.6	0.42	0.00000094
CACNA1H	25	-2.6	4.71	9.83E-20
CILP2	7	-2.6	1.12	0.000000164
ELOVL7	20	-2.6	4.94	3.13E-42
GLRB	17	-2.6	2.4	0.00000417
GNG7	7	-2.6	2.53	1.86E-16
JAKMIP2	7	-2.6	0.14	0.0000101
JAM2	1	-2.6	1.95	4.03E-10
LOC100847777	9	-2.6	0.72	0.000000304
MARCKS	9	-2.6	3.15	3.1E-21
FBXO32	14	-2.7	0.5	0.000482
LRP1B	2	-2.7	3.29	0.0000305
MBOAT2	11	-2.7	0.1	0.00000719
NIPAL1	6	-2.7	1.57	2.57E-10
RIMKLB	5	-2.7	3.55	4.99E-24
SMARCA1	X	-2.7	6.13	2.87E-43
TMTC2	5	-2.7	5.38	8.35E-41
ACTG2	11	-2.8	7.52	6.36E-33
B3GALNT1	1	-2.8	1.14	0.00000996
CCDC158	6	-2.8	0.11	0.000000435
GREM1	10	-2.8	5.54	5.71E-34
LILRB3	18	-2.8	1.58	0.00108
LOC787103	9	-2.8	2.54	2.18E-19
STAC2	19	-2.8	0.56	0.00000157
TMEM119	17	-2.8	6.13	2.37E-30
VWA5A	29	-2.8	2.74	3.08E-08
CRISPLD1	14	-2.9	1.72	0.000000453
FGD4	5	-2.9	2.74	1.3E-21
NEURL1B	20	-2.9	0.65	0.00000601
SRL	25	-2.9	1.64	3.05E-12
TBXA2R	7	-2.9	3.15	1.11E-21
CD274	8	-3	0.22	0.0000401
CDKN2B	8	-3	6.04	7.02E-33
NOG	19	-3	2.06	6.22E-14
SPNS2	19	-3	1	2.63E-08
TRPV2	19	-3	0.12	0.000000198
B3GALT2	16	-3.1	0.94	1.7E-10
GLI1	5	-3.1	0.37	0.0335
PNPLA1	23	-3.1	0.84	0.0000273
AQP1	4	-3.2	7.73	2.41E-20
DPT	16	-3.2	3.91	1.5E-34
MAP2K6	19	-3.2	0.92	3.25E-10

MATN3	11	-3.2	4.17	7.34E-30
PDLIM2	8	-3.2	6.04	2.73E-40
TRAF5	16	-3.2	2.26	3.13E-18
TTC39A	3	-3.2	1.92	1.31E-12
ACTC1	10	-3.3	1.11	8.64E-14
ADAMTS14	28	-3.3	1.32	4.26E-12
CCDC102B	24	-3.3	1.05	6.6E-12
IGF2BP3	4	-3.3	3.29	9.88E-30
LOC522631	24	-3.3	0.26	2.11E-09
SEMA3E	4	-3.3	2.95	7.83E-23
C19H17orf61	19	-3.4	3.3	0.000106
COL8A2	3	-3.4	3.1	9.41E-34
GLUL	16	-3.4	7.98	3.01E-37
GLULP	2	-3.4	3.91	4.26E-55
LOC100299139	24	-3.4	3.47	1.93E-42
PTHLH	5	-3.4	1.72	2.82E-10
SHANK1	18	-3.4	1.13	3.01E-14
AQP11	29	-3.6	0.22	1.81E-11
CHN1	2	-3.6	5.23	7.6E-60
GNAI1	4	-3.7	2.48	0.0000531
THBD	13	-3.7	2.73	8.54E-31
WNT11	15	-3.7	0.73	9.16E-13
FOS	10	-3.8	3.95	5.91E-58
GZMA	20	-3.8	0.32	0.0000223
SCN3A	2	-3.8	1.93	2.36E-09
AGAP2	5	-3.9	0.06	0.000028
FAM171B	2	-4	2.11	3.52E-17
LTBP1	11	-4	6.91	2.38E-61
MGC148692	6	-4	0.56	5.27E-08
MREG	2	-4	2.54	4.62E-20
NKX2-2	13	-4.1	1.42	5.32E-21
SLC7A8	10	-4.1	0.01	5.85E-10
STK31	4	-4.1	0.08	0.000027
TRPS1	14	-4.1	2.02	9.26E-18
FEZ1	29	-4.2	0.96	6.22E-10
ARHGEF26	1	-4.3	1.01	8.64E-19
LGI2	6	-4.4	0.71	0.0000166
LOC517509	10	-4.4	1.65	5.5E-09
PODN	3	-4.4	0.65	1.01E-11
ASPA	19	-4.6	1.4	3.54E-20
IGF2BP2	1	-4.6	2.77	8.87E-20
LMTK3	18	-4.6	1.74	3.3E-14
GRIK5	18	-4.8	0.31	4.07E-11
ITGA8	13	-4.9	3.38	6.57E-49
WNT2B	3	-4.9	0.44	1.11E-08

C16H1orf129	16	-5	0.37	0.00551
DES	2	-5.1	0.4	9.06E-15
SORBS2	27	-5.1	0.14	3.22E-11
PGM5	8	-5.2	2.45	3.55E-40
ATP6AP1L	7	-5.3	3.53	5.85E-40
CTNNA2	11	-5.4	2.54	3.13E-30
TNFSF4	16	-5.4	0.93	5.59E-18
CXCR4	2	-5.6	0.23	5.48E-12
SPOCK1	7	-5.7	2.85	1.28E-19
MUM1L1	X	-5.8	0.35	1.32E-15
BRSK1	18	-5.9	0.66	6.47E-21
LOC100848949	1	-5.9	2.36	5.84E-13
TMEM40	22	-6	1.48	1.46E-25
LOC100848103	25	-6.1	2.79	4.19E-48
MEST	4	-6.1	3.02	3.05E-55
HDAC9	4	-6.3	1.49	1.85E-32
MEGF6	16	-6.5	4.22	3.16E-141
ADRB2	7	-6.6	0.99	4.87E-26
POSTN	12	-7.1	7.41	1.1E-28
SEMA3D	4	-7.1	4.99	1.35E-41
LOC100847414	4	-7.7	4.59	9.98E-43
RSPO2	14	-7.9	0.45	1.16E-15
GDAP1L1	13	-8.4	0.71	7.61E-26
LOC511130	X	-8.9	1.38	0.0000349
LOC784924	4	-9.1	0.27	1.03E-25
LOC532218	5	-10	3.9	0.00096
LOC100847438	14	-10.2	0.55	0.00274
LOC100848767	4	-11.9	1.42	7.9E-46
USP43	19	-11.9	1.1	4E-14
EYA4	9	-21.6	2.73	1.41E-93
CD200R1L	1	-23.1	2.54	1.25E-17
